# Glucose-Dependent Insulin Secretion in Pancreatic β-Cell Islets from Male Rats Requires Ca^2+^ Release via ROS-Stimulated Ryanodine Receptors

**DOI:** 10.1371/journal.pone.0129238

**Published:** 2015-06-05

**Authors:** Paola Llanos, Ariel Contreras-Ferrat, Genaro Barrientos, Marco Valencia, David Mears, Cecilia Hidalgo

**Affiliations:** 1 Institute for Research in Dental Sciences, Facultad de Odontología, Universidad de Chile, Santiago, Chile; 2 Center of Molecular Studies of the Cell, Facultad de Medicina, Universidad de Chile, Santiago, Chile; 3 Physiology and Biophysics Program, Facultad de Medicina, Universidad de Chile, Santiago, Chile; 4 Human Genetics Program, Institute of Biomedical Sciences, Facultad de Medicina, Universidad de Chile, Santiago, Chile; 5 Biomedical Neuroscience Institute, Facultad de Medicina, Universidad de Chile, Santiago, Chile; University of Newcastle, AUSTRALIA

## Abstract

Glucose-stimulated insulin secretion (GSIS) from pancreatic β-cells requires an increase in intracellular free Ca^2+^ concentration ([Ca^2+^]). Glucose uptake into β-cells promotes Ca^2+^ influx and reactive oxygen species (ROS) generation. In other cell types, Ca^2+^ and ROS jointly induce Ca^2+^ release mediated by ryanodine receptor (RyR) channels. Therefore, we explored here if RyR-mediated Ca^2+^ release contributes to GSIS in β-cell islets isolated from male rats. Stimulatory glucose increased islet insulin secretion, and promoted ROS generation in islets and dissociated β-cells. Conventional PCR assays and immunostaining confirmed that β-cells express RyR2, the cardiac RyR isoform. Extended incubation of β-cell islets with inhibitory ryanodine suppressed GSIS; so did the antioxidant N-acetyl cysteine (NAC), which also decreased insulin secretion induced by glucose plus caffeine. Inhibitory ryanodine or NAC did not affect insulin secretion induced by glucose plus carbachol, which engages inositol 1,4,5-trisphosphate receptors. Incubation of islets with H_2_O_2_ in basal glucose increased insulin secretion 2-fold. Inhibitory ryanodine significantly decreased H_2_O_2_-stimulated insulin secretion and prevented the 4.5-fold increase of cytoplasmic [Ca^2+^] produced by incubation of dissociated β-cells with H_2_O_2_. Addition of stimulatory glucose or H_2_O_2_ (in basal glucose) to β-cells disaggregated from islets increased RyR2 *S*-glutathionylation to similar levels, measured by a proximity ligation assay; in contrast, NAC significantly reduced the RyR2 *S*-glutathionylation increase produced by stimulatory glucose. We propose that RyR2-mediated Ca^2+^ release, induced by the concomitant increases in [Ca^2+^] and ROS produced by stimulatory glucose, is an essential step in GSIS.

## Introduction

In the electrically excitable pancreatic β-cells, an increase in intracellular Ca^2+^ concentration ([Ca^2+^]_i_) is the primary trigger for glucose-stimulated insulin secretion (GSIS) [1]. Current models propose that GSIS entails a sequence of events initiated by glucose uptake into β-cells via a low affinity glucose transporter (GLUT-2). The ensuing accelerated metabolism of intracellular glucose increases the cytoplasmic ATP/ADP ratio [2, 3], which sequentially causes plasma membrane depolarization through closure of ATP-sensitive K^+^ channels and Ca^2+^ influx through voltage-dependent Ca^2+^ channels; the resulting increase in [Ca^2+^]_i_ promotes insulin secretion [4].

Previous studies have reported that Ca^2+^ release from intracellular stores contributes to the [Ca^2+^]_i_ rise induced by glucose in β-cells [5]. Both, the inositol 1,4,5-trisphosphate (InsP_3_) receptor [6] and the ryanodine receptor (RyR) [7] channels mediate Ca^2+^ release from sarco/endoplasmic reticulum (SR/ER); these two channel types are present in pancreatic β-cells [8, 9]. The role of InsP_3_ receptor-mediated Ca^2+^ release in muscarinic receptor-stimulated insulin secretion is well-established [10]. In addition, β-cells undergo Ca^2+^-induced Ca^2+^ release (CICR) in response to Ca^2+^ entry through plasma membrane L-type Ca^2+^channels [11–13]. Yet, the contribution of CICR and the specific role of RyR-mediated CICR in GSIS remain undefined.

Mammalian cells express three RyR isoforms (RyR1, RyR2, RyR3) that display ~70% identity and are encoded by three different genes [7]. Physiological ions and endogenous molecules (Ca^2+^, ATP, Mg^2+^ and cyclic ADP-ribose), pharmacological agents, phosphorylation and oxidation reactions modulate RyR channel activity [7]. Studies addressing the participation of RyR-mediated Ca^2+^ release in GSIS have not provided conclusive evidence. Membrane fractions isolated from INS-1 and RINmF5 β-cell lines [14] or from MIN6 β-cells [15] contain all three RyR isoforms; the RyR2 isoform has the highest expression level, although it is expressed at much lower density than in primary hippocampal neurons [14] or muscle cells [15]. Of note, RyR agonists elicit Ca^2+^ release from microsomes isolated from islets [16], or from ER isolated from β-cells [16, 17]. By mediating CICR via PKA-independent signaling mechanisms, in INS-1 rat insulinoma cells RyR channels may contribute to the potentiation of GSIS produced by the hormone glucagon-like peptide 1 (GLP-1) [18]. Other reports have suggested RyR involvement in the [Ca^2+^]_i_ increase produced by glucose or agonists in pancreatic β-cells [14, 16, 17, 19, 20]. Moreover, treatment of the mouse insulinoma cell line MIN6 with inhibitory ryanodine (μM range) decreases GSIS [15]. In contrast, other studies have reported that incubation with inhibitory ryanodine does not prevent insulin secretion in human islets [21] or in the INS-1 rat insulinoma cell line [22]. These conflicting results justify further studies into the role of RyR-mediated Ca^2+^ release on GSIS.

In addition to increasing [Ca^2+^]_i_, glucose stimulates by different cellular pathways the generation of reactive oxygen species (ROS) in β-cells [23]; increased cellular ROS levels regulate physiological [24] and pathophysiological processes [23]. In MIN6 cells, elevated glucose levels and sulfonylureas, which stimulate depolarization by inhibition of ATP-sensitive K^+^ channels, seem to enhance ROS production through NADPH oxidase (NOX) activation [25]. Most studies describing the effects of ROS in β-cells have focused on their deleterious actions when present in excess [26]. Yet, ROS act as intracellular signals for insulin secretion when present at physiological levels [24]. Glucose oxidation under physiological conditions results in hydrogen peroxide (H_2_O_2_) and hydroxyl radical generation [27]. Of note, treatment of rat islets kept at basal glucose concentrations with hydrogen peroxide or alloxan, a molecule which acutely increases intracellular H_2_O_2_ levels, causes a rapid elevation of [Ca^2+^]_i_ and produces a transient increase in insulin release [28, 29].

In other cell types, ROS stimulate RyR-mediated CICR [30]. Given the proposed role of ROS as physiological signals in GSIS [24, 31], plus the redox-sensitivity of RyR-mediated CICR, we hypothesized that glucose, by inducing an initial [Ca^2+^]_i_ increase due to Ca^2+^ entry and increasing cellular ROS levels, promotes RyR-mediated CICR via RyR redox modifications; the resulting amplification of Ca^2+^ entry signals would promote GSIS. Our results support this hypothesis, since a stimulatory glucose concentration generated ROS that increased RyR *S*-glutathionylation, while RyR inhibition or the antioxidant N-acetyl cysteine (NAC) significantly decreased or abolished GSIS. The main findings of this work were previously presented in abstract form (Biological Research 2009, 42 (Supplement A), R-115).

## Materials and Methods

### Reagents

All reagents used were of analytical grade. Caffeine, NAC, polylysine, RPMI 1640 culture medium and carbamylcholine chloride (carbachol, CCh) were from Sigma-Aldrich Chemical (St Louis, MO). Fura-2 acetoxymethyl ester (fura-2-AM), Fluo-4 acetoxymethyl ester (Fluo-4-AM), 5-(6)-chloromethyl-2',7'-dichlorodihydro-fluorescein diacetate acetyl ester (CM-H_2_DCFDA), Dispase-EDTA, Dulbecco modified Eagle’s medium, BODIPY-FL-X Ryanodine (BODIPY-Rya) and Calcium Calibration Kit 1 with Magnesium were from Invitrogen (Eugene, OR). Ryanodine was from Alexis Biochemical (Farmingdale, NY), and H_2_O_2_ from Merck (Whitehouse Station, NJ). The Duolink II red starter kit was from Olink-Bioscience (Uppsala, Sweden).

### Antibodies

Anti-insulin antibodies were from Dako (Carpinteria, CA), anti-RyR2 from Affinity BioReagents (Golden, CO) or Millipore Corp. (Billerica, MA) and anti-calnexin from Sigma (St Louis, MO). The secondary antibodies used were anti-guinea pig FITC from Jackson Immuno Research (West Grove, PA). Alexa Fluor 635 anti-mouse IgG, and Alexa Fluor 635 anti-rabbit IgG, were both from Invitrogen (Eugene, OR). Antibodies against *S*-glutathionylated protein adducts were from Virogen Corp. (Watertown, MA).

### Animals

Male Sprague-Dawley rats weighing 250–300 g (45–60 days old) were obtained from the Central Animal Facility of the Faculty of Medicine, Universidad de Chile. The animals were kept at 23°C under a 12 h light–dark cycle, with free access to food and water. The Bioethics Committee for Animal Research, Faculty of Medicine, Universidad de Chile, approved all experimental protocols used in this work.

### Rat Pancreatic Islet Isolation

The pancreas extracted from male rats was digested with collagenase to isolate the islets of Langerhans as previously described [32]. Islets were picked by hand under a dissecting microscope, rinsed three times in Hanks solution and cultured overnight in an incubator under 95% O_2_/5% CO_2_. The RPMI 1640 culture medium was supplemented with 5 mM glucose, 10% fetal bovine serum (FBS), 10 mM HEPES, 100 units/ml penicillin, 100 μg/ml streptomycin. Cell viability was evaluated as described in detail elsewhere [33].

### Isolation of Pancreatic β-Cells

For studies on single cells, islets were dispersed into cell suspensions by digestion with dispase-EDTA. The digested suspension was subsequently triturated by passage through a fire-polished Pasteur pipette. Cells suspended in RPMI 1640 containing 10% FBS and 5 mM glucose were plated onto glass coverslips coated with 40 μg/ml polylysine to facilitate cell adherence.

### MIN6 Cell Culture

The mouse insulinoma MIN6 pancreatic β-cell line [34], kindly provided by Dr. Paolo Meda (Geneva, Switzerland), was maintained at 37°C in Dulbecco’s modified Eagle’s medium containing 15% FBS, 100 mU/ml penicillin, 100 mg/ml streptomycin, 11 mM glucose, in an atmosphere of 95% O_2_/5% CO_2_.

### Insulin Secretion

All determinations of insulin secretion were performed in pancreatic islets under static incubation. Briefly, 15 islets of 150–200 μm diameter kept in 24-well plates were pre-incubated for 1 h at 37°C in Krebs–Ringer bicarbonate (KRB) buffer containing (in mM): 120 NaCl, 5 KCl, 1 MgCl_2_, 2.5 CaCl_2_, 25 NaHCO_3_ (equilibrated with 5% CO_2_−95% O_2_, pH 7.4), 0.5% bovine serum albumin, 2.8 mM glucose. The pre-incubation medium was then replaced with KRB buffer supplemented with different glucose concentrations (basal: 2.8 mM; stimulatory: 16.7 or 27.7 mM) as well as other test agents. After incubation for 1 h at 37°C, the supernatant was collected and stored at -80°C for later analysis of insulin content by ELISA (Mercodia Rat Insulin ELISA, Sweden).

### Evaluation of ROS Production in β-Cells and Pancreatic Islets

The commercial probe CM-H_2_DCFDA was used to evaluate intracellular ROS generation. Cells or islets were placed on glass coverslips and cultured overnight in RPMI 1640 containing 10% FBS and 5 mM glucose. The coverslips were then washed with Hank´s buffer solution (HBSS; in mM: 125 NaCl, 5.4 KCl, 5 NaHCO_3_, 0.3 Na_2_HPO_4_, 0.4 KH_2_PO_4_, 5.5 glucose, 10 HEPES-Na; pH 7.4), and incubated for 1 h at 37°C with KBR buffer containing 2.8 mM glucose, 16.7 mM glucose or 2.8 mM glucose plus 100 μM H_2_O_2_. Cells were loaded next with 10 μM CM-H_2_DCFDA and after 60 min digital fluorescence images were obtained in a confocal microscope (Pascal 5, Zeiss, Germany), using an excitation wavelength of 488 nm and a 515 nm long pass emission filter.

### [Ca^2+^]_i_ Measurements

Isolated β-cells were maintained on glass coverslips overnight prior to each experiment. Cells were loaded with the Ca^2+^-sensitive dye fura-2 AM (2 μM with 0.02% Pluronic acid in HBSS) by incubation for 45 min at 37°C. To test the effects of H_2_O_2_, cells were incubated for 1 h with 100 μM H_2_O_2_ and then loaded with fura-2 AM for 30 min. All fluorescence determinations were performed at room temperature. Dual wavelength excitation microspectrofluorimetry was performed ratiometrically at 1-s intervals using a digital video imaging system (Ionwizard 4.4; IonOptix Corp., Milton, MA, USA). Calibration of raw fluorescence values was performed using fura-2 pentapotassium salt dissolved in calibration buffer solutions (Calcium Calibration Kit 1 with Magnesium). Solutions containing H_2_O_2_ were prepared each time just prior to use.

To evaluate ER Ca^2+^ content, we inhibited the SERCA pump by adding thapsigargin in Ca^2+^-free solution, and monitored with Fluo-4 (K_d_ = 345 nM) the cytoplasmic Ca^2+^ signals arising from the ensuing net Ca^2+^ efflux from the ER. To this purpose, isolated β-cells were pre-incubated for 30 min at 37°C with 5 μM Fluo-4-AM (with 0.02% Pluronic acid in HBSS). After washing isolated β-cells for 10 min in modified HBBS solution to allow complete dye de-esterification, cultures were transferred to Ca^2+^-free medium just prior to fluorescence recording. Fluorescence images of cytoplasmic Ca^2+^ signals were obtained at 1-s intervals with an inverted confocal microscope (Carl Zeiss, Axiovert 200, LSM 5 Pascal, Oberkochen, Germany, Plan Apochromatic 63x Oil DIC objective, optical slice 1000 μm, excitation 488 nm, argon laser beam). Image data were acquired from different regions of optical interest (ROI) defined with the same area and located in the cell bodies, excluding the nucleus; frame scans were averaged using the equipment data acquisition program. All experiments were done at room temperature (20–22°C).

### Binding of BODIPY FL-X Ryanodine

Binding of BODIPY FL-X ryanodine to pancreatic islets was evaluated by confocal microscopy. The islets were loaded with 50 μM BODIPY FL-X ryanodine for 1 or 12 h at 37°C and then washed with KRB three times and maintained in this solution. Digital images of BODIPY FL-X fluorescence were acquired in a confocal microscope (Pascal 5, Zeiss, Germany) using an excitation wavelength of 488 nm and a 515 nm long-pass emission filter.

### Immunofluorescence Staining

Pancreatic β-cells or MIN6 cells grown on coverslips were fixed in phosphate-buffered saline (PBS; in mM: 137 NaCl, 2.7 KCl, 8 Na_2_HPO_4_, 1.46 KH_2_PO_4_; pH 7.4) containing 3% formaldehyde at room temperature for 15 min. Cells were treated next with 0.25% Triton X-100 in PBS for an additional 15 min, and incubated with anti-insulin, anti-RyR2 or anti-calnexin antibodies. Anti-guinea pig FITC, Alexa Fluor 635 anti-mouse IgG or Alexa Fluor 635 anti-rabbit IgG were used as secondary antibodies. Nuclei were stained with Hoechst as described elsewhere [35]. The cross sections of pancreatic tissue were 5 μm thick.

### *In situ* Proximity Ligation Assay (PLA)

To detect RyR2 *S*-glutathionylation *in situ*, we used a proximity ligation assay (Duolink II red starter kit) according to the manufacturer instructions, plus primary antibodies against RyR2 (Millipore Corp.) and *S*-glutathionylated protein adducts. Briefly, β-cells disaggregated from islets and incubated 24 h in RPMI 1640 culture medium containing 10% FBS and 5 mM glucose, were incubated overnight at 4°C in a humid chamber with the above primary antibodies. Cells were incubated next for 1 h at 37°C with Duolink, plus and minus secondary antibodies; these secondary antibodies contain oligonucleotides that in Duolink Ligation Solution form a closed circle when in close proximity (optimal resolution, 30–40 nm). Circle formation was detected by subsequent addition of polymerase to amplify the closed circles, which were detected next with the complementary oligonucleotides, fluorescently labeled, provided in the Duolink kit. Fluorescence images were acquired in a confocal microscope as described above. After incubation with the PLA probes, β-cells were identified by immunofluorescence with insulin antibodies.

*Statistical analysis*-Data are expressed as Mean ± SEM. One-way ANOVA followed by Tukey's multiple comparison test was used to compare groups. A p-value ≤ 0.05 was considered significant.

## Results

### Pancreatic Islet β-cells Express the RyR2 Isoform

**Fig 1:**
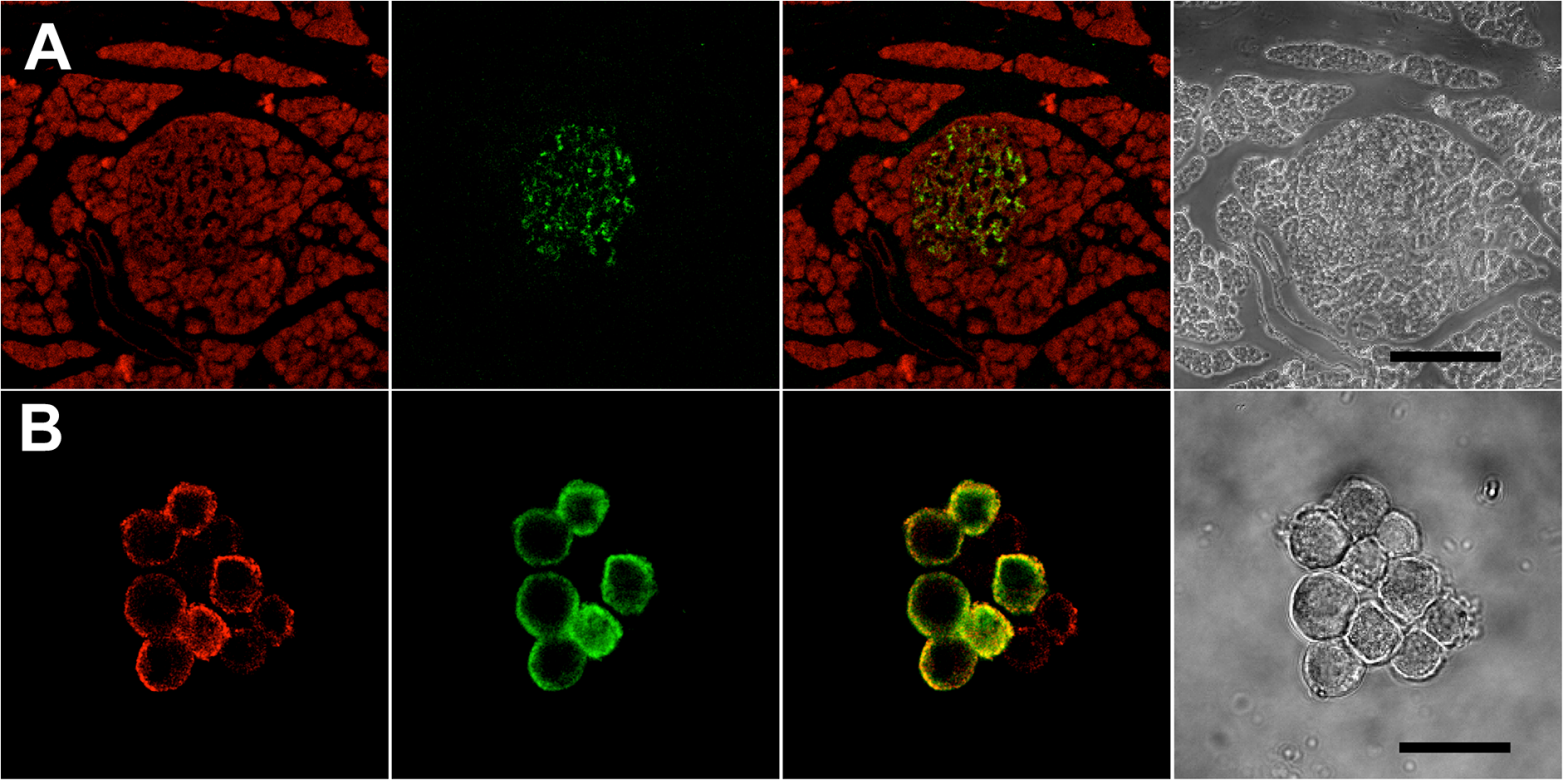
Localization of RyR2 in rat pancreas and rat pancreatic β-cells. (A) The panel shows a representative image of a rat pancreatic tissue section stained for RyR2 (red) and insulin (green) as a marker of pancreatic β-cells; overlapping of images at right shows the presence of both proteins in the center of the islet (islet of Langerhans). (B) The panel shows cells from a dispersed islet. Insulin staining is shown in green and RyR2 in red. Overlapping of images shows the presence of RyR2 in pancreatic β-cells as well as in other cell types of the islet. The calibration bars represent 200 μm in (A), 20 μm in (B).

Previous reports indicate that β-cell lines express the three mammalian RyR isoforms [14, 15], plus a newly described RyR isoform [36]. By immunohistochemical analysis, we detected the presence of the cardiac RyR2 isoform in rat endocrine pancreas. In cross sections of pancreatic tissue, RyR2 fluorescent label was present in islets (endocrine pancreas) and pancreatic acini (exocrine pancreas) (Fig 1A). Within the islets, the RyR2 signal co-localized with insulin, a specific marker of pancreatic β-cells. In disaggregated islets, immunostaining for RyR2 was apparent in both insulin-positive and insulin-negative cells (Fig 1B). The RyR2 signal in β-cells, which have a highly developed ER typical of secretory cells, was strongest in the cell periphery near the plasma membrane. By immunocytochemical analysis, we also detected RyR2 in the mouse pancreatic β-cell line MIN6 and in pancreatic β-cells dissociated from islets. In both cell types, the RyR2 signal co-localized with calnexin (S1 Fig), a well known ER marker [37]. Immunoblot analysis of MIN6 cell homogenates revealed a distinct band corresponding to RyR2 (S2 Fig). In contrast, we did not detect a band corresponding to RyR2 in immunoblots of islet homogenates. Presumably, RyR2 density in whole islet homogenates is too low for detection by this technique; this feature would explain why there are no reports in the literature describing the presence of RyR2 in islets by immunoblot analysis. Taken together, these results confirm that pancreatic β-cells express the RyR2 protein isoform, which seems to be the predominant RyR isoform present in β-cells [9, 14]. We did not examine the presence of other RyR isoforms. Additionally, semi-quantitative RT-PCR analysis showed that rat pancreatic islets expressed RyR2 mRNA (S2 Fig), confirming previous findings [16, 17, 38].

### Equilibration of a Fluorescent Ryanodine Analog in Pancreatic β-Cell Islets

Ryanodine is a plant alkaloid that acts as a RyR channel agonist at nM concentrations but is a potent and highly selective channel inhibitor at μM concentrations. Because of these distinctive actions and its high degree of specificity (to date no other cellular targets have been reported), ryanodine is widely considered the “gold standard” to test RyR channel function and is often used to functionally identify RyR channels [7]. Ryanodine is membrane permeable, so within cells it targets ER-resident RyR channels where it binds preferentially to RyR channels in the open state. Hence, effective inhibition of RyR channels present in complex systems, such as the pancreatic β-cell islets, is likely to require both high concentrations of ryanodine and long incubation times to ensure access of inhibitory ryanodine concentrations to all cells within the islet. To test if incubation time affected the distribution of ryanodine, rat islets were incubated for 1 h or 12 h with BODIPY-ryanodine, a permeable and fluorescent ryanodine analog. BODIPY-ryanodine showed a relatively homogeneous distribution throughout the islet after prolonged incubation (12 h; S3B Fig); in contrast, after 1 h of incubation the fluorescent probe was found only in cells present at the periphery of the islet (S3A Fig). Accordingly, we tested below the inhibitory effects of ryanodine on GSIS after incubating islets for 12 h with this plant alkaloid. As detailed below, this long incubation period with inhibitory ryanodine did not prevent insulin secretion in response to carbachol plus stimulatory glucose concentration.

### Glucose-Stimulated Insulin Secretion Requires Functional RyR

**Fig 2:**
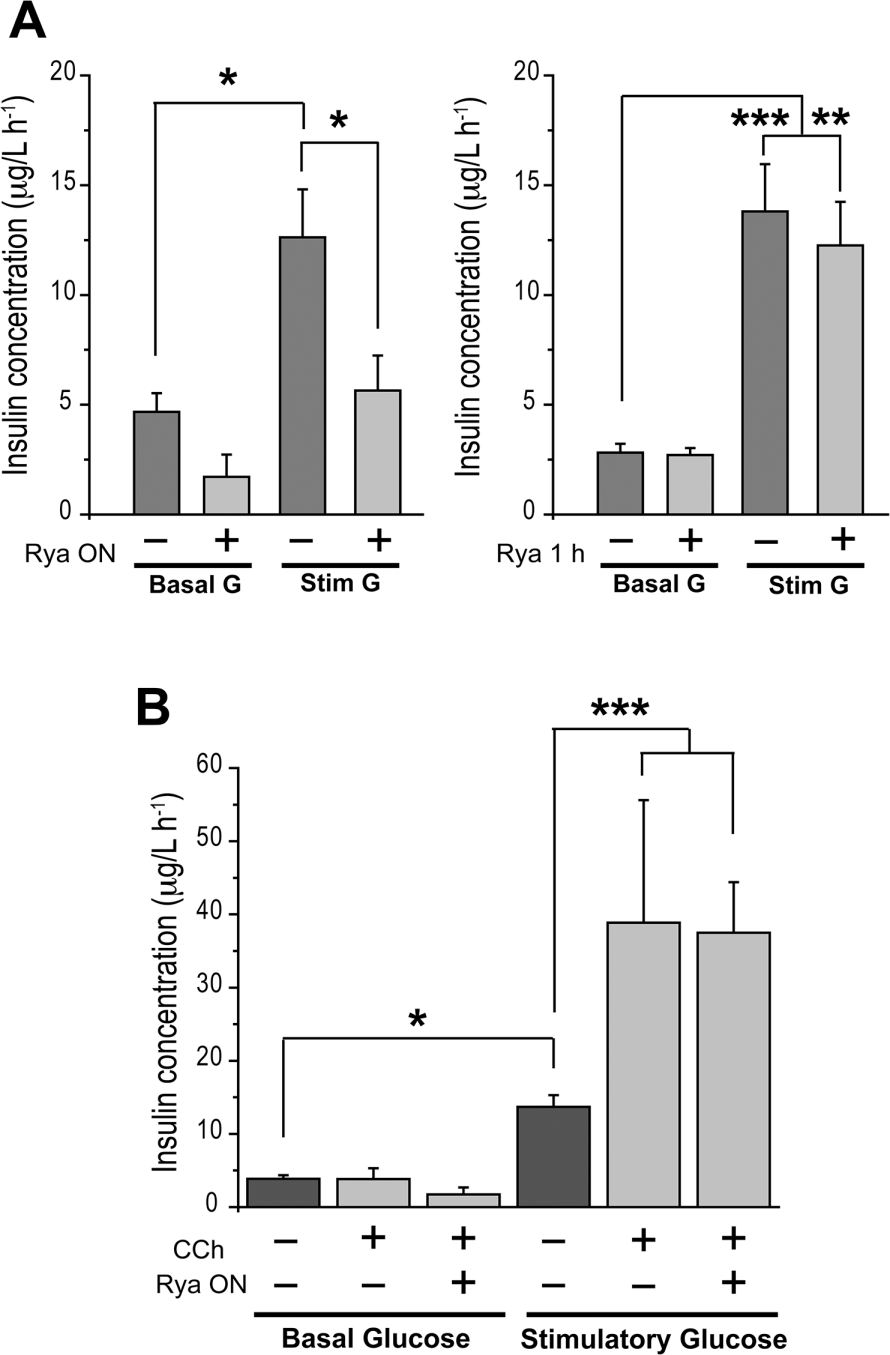
Overnight incubation of pancreatic islets with 200 μM ryanodine inhibits insulin secretion stimulated by glucose but not by glucose plus carbachol. Insulin secretion was determined in groups of 15 islets after incubation for 1 h at 37°C in basal (2.8 mM) or stimulatory glucose (16.7 mM). (A, left) Rya ON: islets were pre-incubated with 200 μM ryanodine for 12 h before determination of insulin secretion after 1 h incubation in ryanodine-free solutions. (A, right) Rya 1 h: islets were pre-incubated with 100 μM ryanodine for 1 h before determination of insulin secretion after 1 h incubation in ryanodine-free solutions; G: glucose. (B) CCh: 30 μM carbachol was added during the 1 h incubation period used to measure insulin secretion. All data represent Mean ± SEM; N = 3 experiments (each condition in triplicate). Statistical significance was determined with one-way ANOVA followed by Tukey multiple comparison test. *: p <0.05; **: p <0.01; ***p <0.001.

Stimulatory glucose (16.7 mM) increased insulin secretion rate (μg/l h^-1^) from an average basal value of 4.7 ± 0.7 to a value of 12.6 ± 2.1 (Fig 2A, left panel). Incubation with inhibitory ryanodine for 12 h decreased GSIS rate to 5.6 ± 1.6 (μg/l h^-1^), a value not significantly different to the average basal level determined in the absence of ryanodine. After 12 h incubation with ryanodine, the average insulin secretion rate in basal glucose (2.8 mM) was 1.7 ± 1.0 (μg/l h^-1^) (Fig 2A, left panel), not significantly different from the average basal value. In agreement with the lack of penetration of BODIPY-ryanodine into the islet after 1 h, pre-incubation with inhibitory ryanodine for 1 h did not affect insulin secretion from islets incubated with basal (2.8 mM) or stimulatory (16.7 mM) glucose compared to controls (Fig 2A, right panel).

To test if islets remained functional and with the ER loaded with Ca^2+^ after prolonged incubation (12 h) with 200 μM ryanodine, we treated islets with 30 μM carbachol to stimulate insulin secretion. Previous reports have established that carbachol, a pharmacological agonist of muscarinic receptors, stimulates insulin secretion from pancreatic β-cells in a strictly glucose-dependent manner, through a pathway that engages Ca^2+^ release mediated by InsP3 receptors [39, 40]. As expected, carbachol did not stimulate insulin secretion when added at basal glucose concentration, but at stimulatory glucose concentration it significantly increased insulin secretion, from 13.7 ± 1.6 to 38.9 ± 16.7 (μg/l h^-1^) (Fig 2B). Joint addition of glucose and carbachol to islets pre-incubated 12 h with inhibitory ryanodine produced insulin secretion rates of 37.5 ± 6.9 (μg/l h^-1^). These values are not significantly different to those produced by carbachol plus glucose in the absence of ryanodine, indicating that inhibitory ryanodine did not affect carbachol-mediated pathways. In addition, by using thapsigargin to inhibit the SERCA pump in Ca^2+^-free solution, and thus promote net Ca^2+^ efflux from the ER, we tested directly if prolonged incubation with inhibitory ryanodine promoted ER depletion. Both control and ryanodine-treated isolated β-cells exhibited similar Ca^2+^ signals in response to thapsigargin addition (S4 Fig), strongly suggesting that ryanodine-treated β-cells had similar ER Ca^2+^ contents as control cells, even after overnight incubation with 200 μM ryanodine. Moreover, ryanodine-treated islets displayed similar ROS levels as controls (S4 Fig), indicating that RyR inhibition did not modify basal ROS production.

### Glucose Stimulates ROS Production in Isolated Islets and Single Pancreatic β-Cells

**Fig 3:**
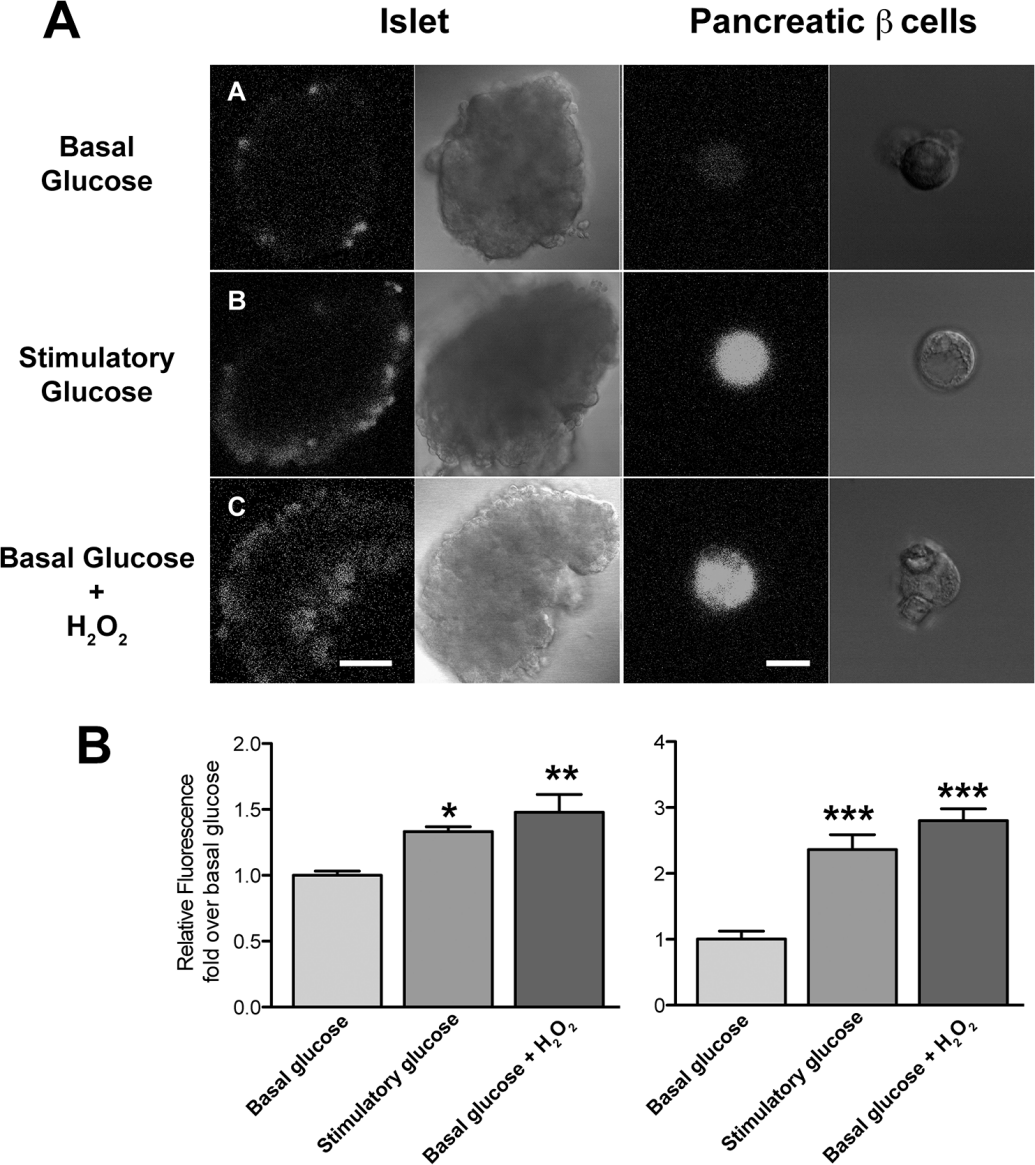
Incubation with exogenous H_2_O_2_ or glucose increases ROS generation. ROS accumulation was detected by confocal microscopy in islets or cells loaded with CM-H_2_DCFDA (10 μM), which cytoplasmic esterase enzymes convert to the redox-sensitive fluorescence reporter H_2_DCFDA. Representative images correspond to isolated islets or pancreatic β-cells incubated for 1 h in Krebs bicarbonate buffer containing: (A) 2.8 mM glucose; (B) 16.7 mM glucose; (C) 2.8 mM glucose plus 100 μM H_2_O_2_ and (D) Quantification of ROS production with the redox-sensitive fluorescence reporter H_2_DCFDA. Similar results were obtained in three independent experiments. Scale bars: 50 μm, islet; 10 μm, β-cell. Statistical significance was determined with one-way ANOVA followed by Tukey multiple comparison test. *: p <0.05; **: p <0.01; ***p <0.001.

In islets and single β-cells loaded with the ROS-sensitive probe CM-H_2_DCF, stimulatory glucose (16.7 mM) increased probe fluorescence 1.3 fold and 2.5-fold, respectively, relative to the basal condition (Fig 3). These results confirm previous reports that glucose increases ROS generation in islets and β-cells [24]. Incubation with H_2_O_2_ for 1 h of islets or β-cells maintained in basal glucose concentration (2.8 mM) also increased probe fluorescence, 1.4 fold in islets and 2.8-fold in cells relative to the basal condition, indicating that H_2_O_2_ addition in basal glucose produces a comparable increase in probe fluorescence as that produced by stimulatory glucose.

### N-Acetyl Cysteine Suppresses GSIS and Inhibits Insulin Secretion Stimulated by Glucose and Caffeine

**Fig 4:**
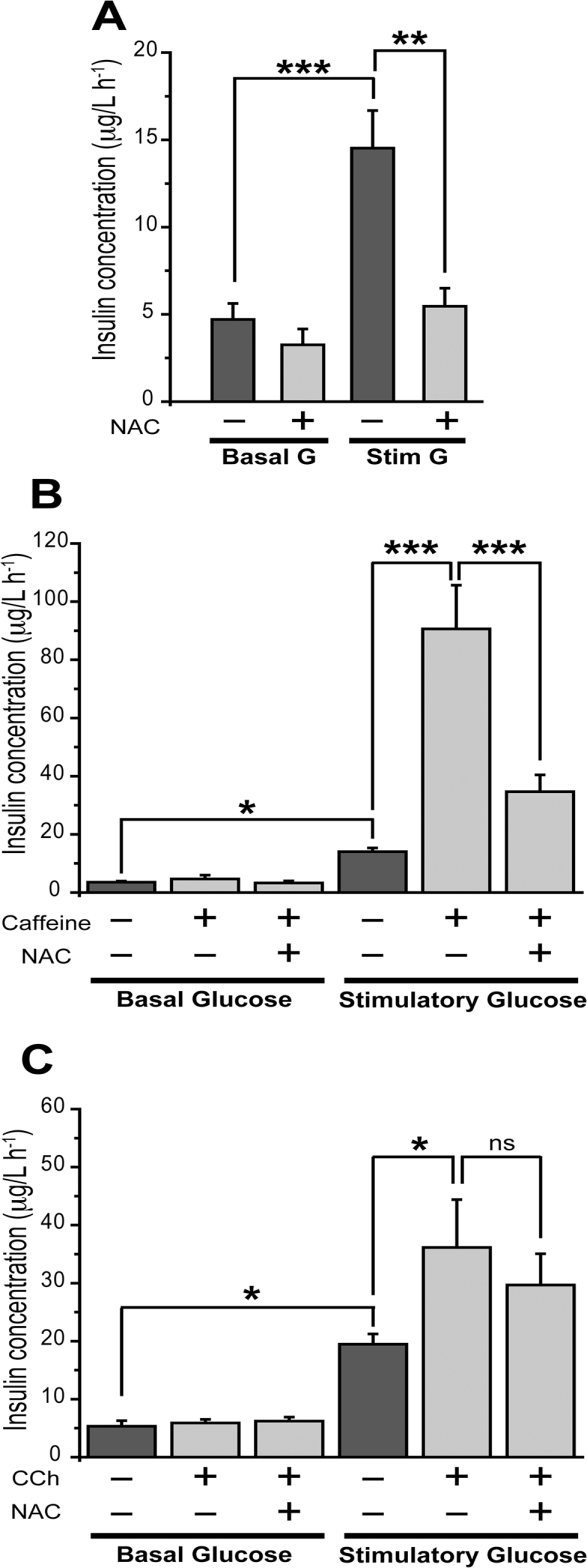
N-acetyl cysteine (NAC) inhibits insulin secretion stimulated by glucose or caffeine but not by carbachol. Islets were pre-incubated at 37°C for 1 h in Krebs bicarbonate buffer supplemented with 2.8 mM glucose in the presence or absence of 10 mM NAC. (A) The effects of NAC on insulin secretion were determined in groups of 15 islets incubated for 1 h at 37°C in basal (2.8 mM) or stimulatory glucose (16.7 mM). Values represent Mean ± SEM; N = 6 experiments. (B) When indicated, caffeine (2.5 mM) was added throughout this second incubation period. Values represent Mean ± SEM; N = 3 experiments. (C) Carbachol was added at a concentration of 30 μM throughout the second incubation period. Values represent Mean ± SEM; N = 3–6 experiments. Statistically significant differences were determined with one-way ANOVA followed by Tukey multiple comparison test. *: p <0.05; **: p <0.01; ***: p <0.001; ns: no significant differences.

Pre-incubation with the antioxidant NAC for 1 h did not affect basal insulin secretion but fully inhibited GSIS, which decreased from 14.6 ± 2.1 to 5.5 ± 1 (μg/l h^-1^) (Fig 4A). Addition of 2.5 mM caffeine, which at this concentration acts primarily as a pharmacological RyR agonist [22], did not stimulate insulin secretion when measured at basal glucose levels (Fig 4B). As reported earlier [41], caffeine markedly stimulated insulin secretion, from 14.0 ± 1.3 to 90.6 ± 15.0 (μg/l h^-1^) when tested at a stimulatory glucose concentration, whereas NAC significantly decreased insulin secretion jointly stimulated by glucose and caffeine (Fig 4B). In contrast, incubation with NAC did not affect insulin secretion jointly stimulated by carbachol plus 16.7 mM glucose (Fig 4C) or by 27.7 mM glucose (S5 Fig).

### Exogenous H_2_O_2_ Has a Dual Effect on Insulin Secretion

**Fig 5:**
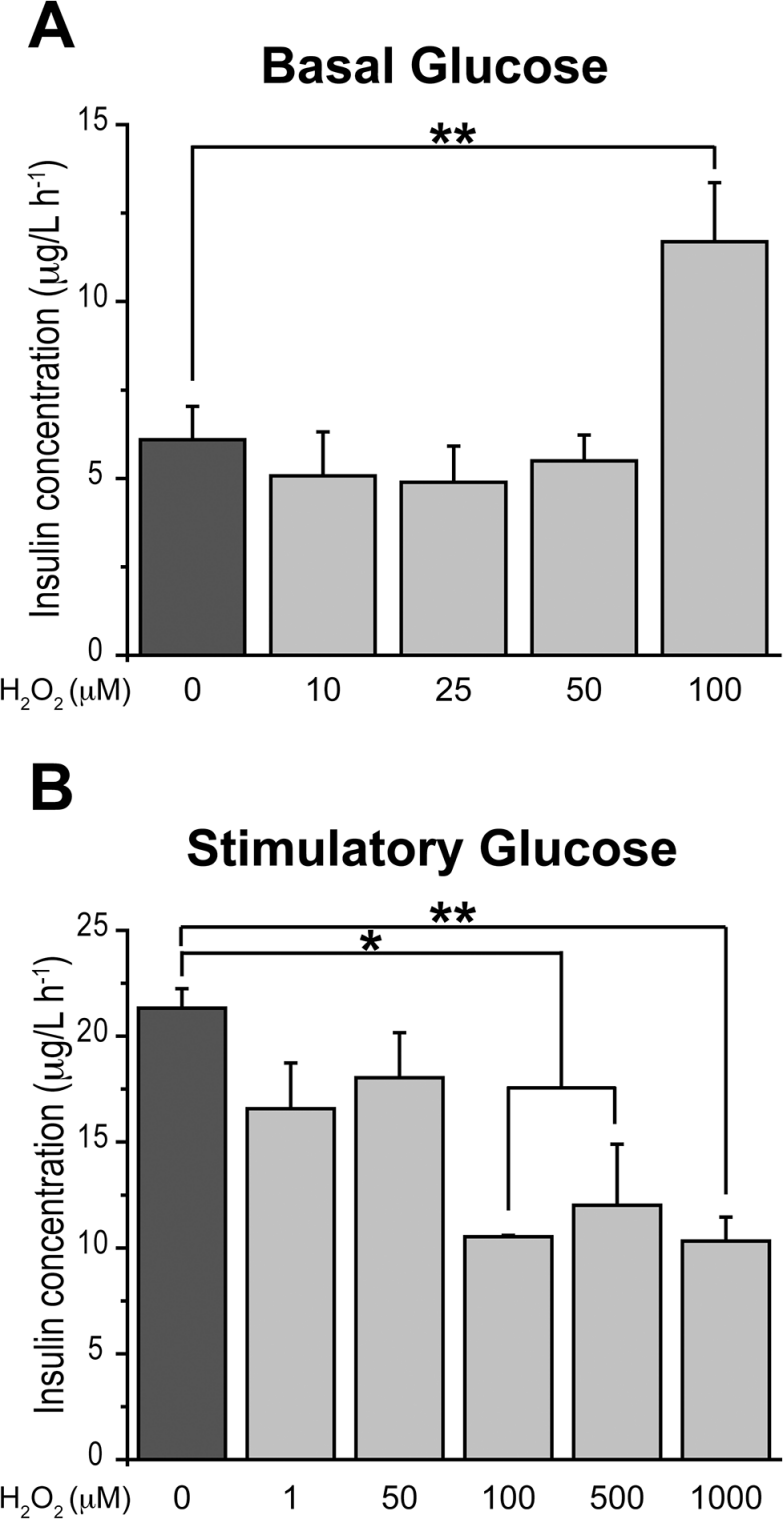
H_2_O_2_ has dual effects on glucose-induced insulin secretion. (A) Insulin secretion in basal glucose (2.8 mM) was determined in islets incubated for 1 h with different concentrations of H_2_O_2_. An increase in insulin secretion in basal glucose was observed at 100 μM H_2_O_2_. (B) Insulin secretion in stimulatory glucose (16.7 mM) in islets incubated for 1 h with different concentrations of H_2_O_2_. Mean ± SEM, N = 3–5. Statistical significance was determined with one-way ANOVA followed by Tukey multiple comparison test. *: p <0.05; **: p <0.01.

Pre-incubation of pancreatic islets for 1 h with H_2_O_2_ added as an exogenous ROS source had a dual effect on insulin secretion. Under conditions of low glucose (2.8 mM), addition of H_2_O_2_ stimulated insulin secretion to a value of 11.7 ± 1.7 (μg/l h^-1^); this value is nearly 2-fold higher than the basal value of 6.1 ± 0.9 (μg/l h^-1^) (Fig 5A). Pre-incubation with 100 μM H_2_O_2_ for 1 h of islets kept in low glucose produced a modest decrease (13%) in cell viability. Under conditions of stimulatory glucose (16.7 mM) concentrations of H_2_O_2_ ≥ 100 μM significantly decreased insulin secretion (Fig 5B); these results are in agreement with a previous report showing that 200 μM H_2_O_2_ significantly decreased GSIS in islets [29]. Concentrations of H_2_O_2_ < 100 μM were ineffective either at basal or stimulatory glucose concentrations.

### Insulin Secretion Induced by H_2_O_2_ at Basal Glucose Concentration Requires Functional RyR Channels

**Fig 6:**
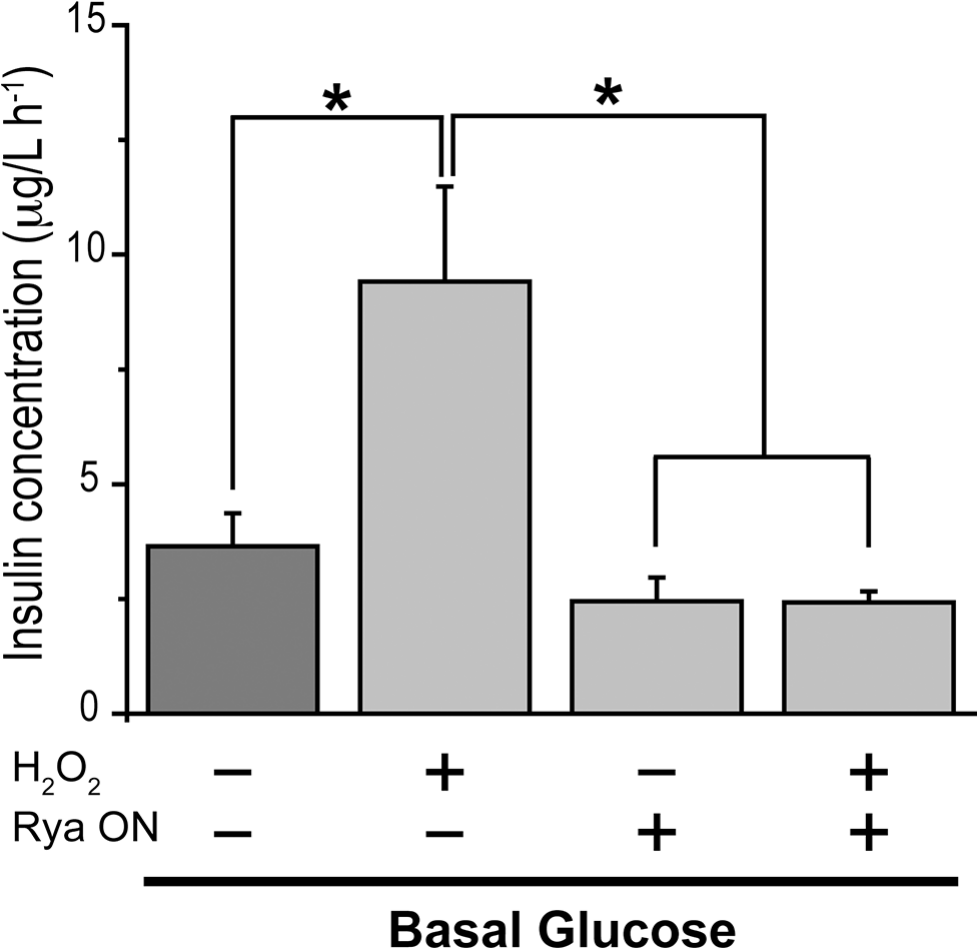
RyR inhibition prevents H_2_O_2_-dependent insulin secretion. Islets were pre-incubated for 1 h at 37°C in Krebs bicarbonate buffer supplemented with 2.8 mM glucose. Groups of 15 islets were then incubated for 1 h at 37°C in the presence or absence of 100 μM H_2_O_2_ in basal glucose (2.8 mM) to measure insulin secretion. Rya ON: islets were pre-incubated with 200 μM ryanodine for 12 h before the 1 h incubation period used to measure insulin secretion. Values represent Mean ± SEM; N = 3. Statistical significance was determined with one-way ANOVA followed by Tukey multiple comparison test. *: p <0.05.

To test RyR participation in the enhancement of insulin secretion induced by H_2_O_2_ in basal glucose concentration, we incubated islets with inhibitory ryanodine for 12 h prior to H_2_O_2_ addition. In these conditions, addition of H_2_O_2_ in basal glucose did not stimulate insulin secretion (Fig 6). In contrast, as illustrated in Fig 6, H_2_O_2_ stimulated insulin secretion > 2-fold in islets kept in basal glucose and not treated with ryanodine, while islets incubated for 12 h with inhibitory ryanodine had comparable levels of insulin secretion (2.4 ± 0.2 μg/l h^-1^) as islets kept in basal glucose (3.4 ± 0.7 μg/l h^-1^).

### RyR-Mediated Ca^2+^ Release Underlies the [Ca^2+^]_i_ Increase Produced by H_2_O_2_

**Fig 7:**
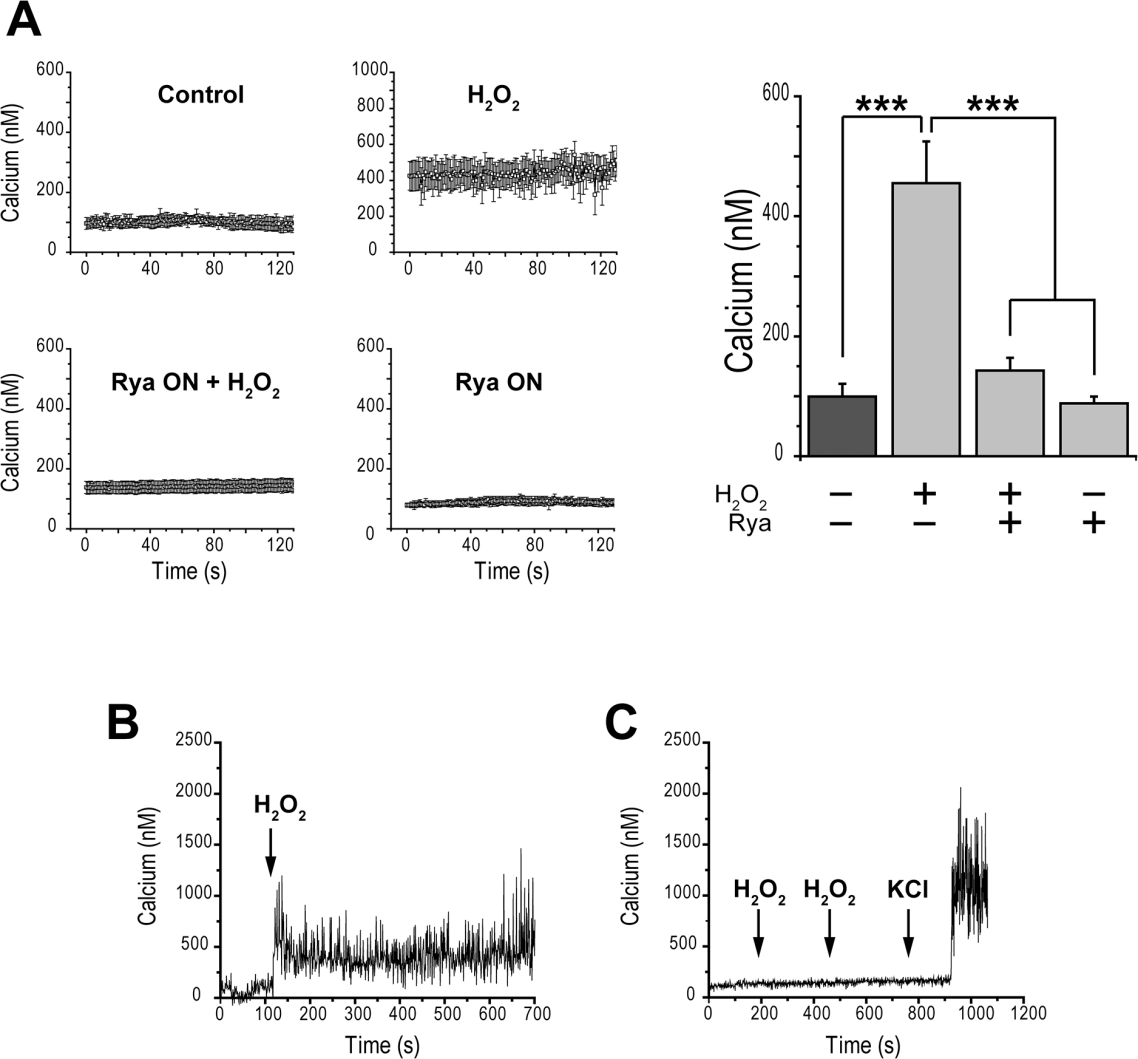
Incubation with exogenous H_2_O_2_ increases [Ca^2+^]_i_ in pancreatic β-cells via activation of RyR-mediated Ca^2+^ release. (A) Records of [Ca^2+^]_i_ vs time obtained from rat pancreatic β-cells pre-incubated for 1 h with 2 μM fura-2-AM in Hanks basal solution (2.8 mM glucose). Control: cells were kept in basal Hanks solution. H_2_O_2_: cells were pre-incubated for 1 h with 100 μM H_2_O_2_ in basal Hanks solution. H_2_O_2_ + Rya ON: cells were pre-incubated with 200 μM ryanodine (Rya) for 12 h and were then incubated for 1 h with 100 μM H_2_O_2_ (in ryanodine-free solution) prior to recording in basal Hanks solution (H_2_O_2_ free). Rya ON: cells were pre-incubated with 200 μM ryanodine for 12 h. At right, quantification of these results, given as Mean ± SEM, N = 3–7. Statistical significance was determined with one-way ANOVA followed by Tukey multiple comparison test. ***: p <0.001. (B) Average record (N = 3) of Ca^2+^ signals elicited by 100 μM H_2_O_2_ in the absence of ryanodine. (C) Average record (N = 3) of Ca^2+^ signals registered in cells pre-incubated with 200 μM ryanodine for 12 h (Rya ON); 100 μM H_2_O_2_ or 90 mM KCl were added in succession, as indicated by the arrows.

Addition of H_2_O_2_ stimulates RyR-mediated CICR in other cell types [30]. The results illustrated in Fig 6 led us to hypothesize that addition of H_2_O_2_ activates RyR-mediated Ca^2+^ release in pancreatic β-cells; the resulting increase in [Ca^2+^]_i_ would cause the increase in insulin secretion induced by H_2_O_2_. To test this hypothesis, we measured [Ca^2+^]_i_ with the fluorescent probe fura-2 (Fig 7A). Incubation for 1 h of disaggregated β-cells with H_2_O_2_ increased [Ca^2+^]_i_ from a basal level of 99.7 ± 21 nM to 455.2 ± 69.6 nM. Cells pre-incubated with inhibitory ryanodine for 12 h displayed an average value of [Ca^2+^]_i_ = 142.6 ± 21.5 nM, which did not change after addition of H_2_O_2_ (Fig 7A). As illustrated in Fig 7B, H_2_O_2_ addition to control cells increased [Ca^2+^]_i_ rapidly (within 10 s) to a value of 324 ± 5.4 nM (mean value, first minute after H_2_O_2_ addition, N = 3). This increase occurred as a consequence of RyR-mediated Ca^2+^ release since overnight incubation with inhibitory ryanodine prevented the fast [Ca^2+^]_i_ increase produced by H_2_O_2_ (Fig 7C). Yet, these same cells did respond to subsequent addition of 90 mM KCl with a marked increase in [Ca^2+^]_i_ (Fig 7C). The observations that disaggregated β-cells incubated overnight with inhibitory ryanodine maintained [Ca^2+^]_i_ at resting levels, and responded to KCl, show that Ca^2+^ homeostasis and depolarization-induced Ca^2+^ influx through voltage-gated Ca^2+^ channels remained largely unaffected by this treatment.

### Glucose-Dependent ROS Production Increases *S*-glutathionylation of RyR Cysteine Residues

**Fig 8:**
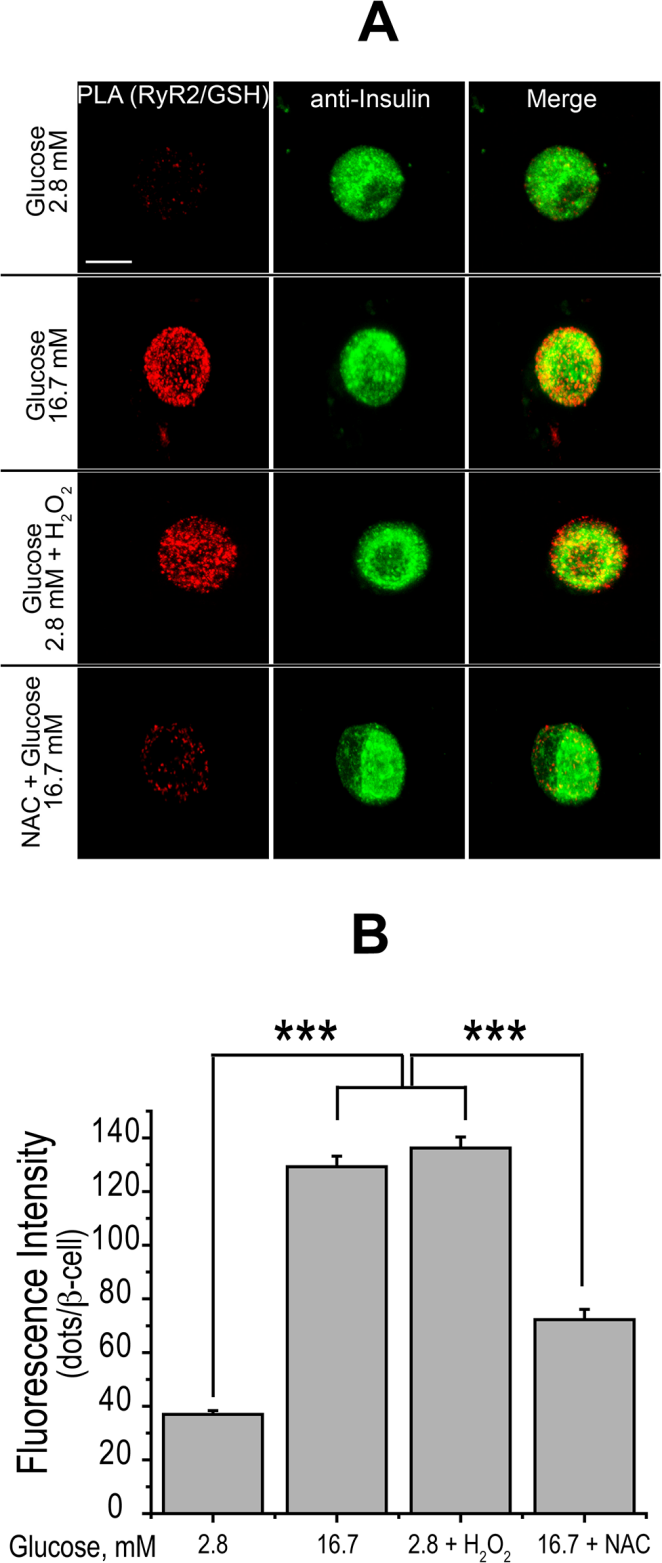
Stimulatory glucose concentrations and H_2_O_2_ promote RyR2 *S*-glutathionylation; NAC inhibits this response. (A) Representative image of **β**-cells disaggregated from islets showing RyR2 *S*-glutathionylation with the PLA assay (red fluorescence) and insulin immunostaining (in green). H_2_O_2_: 100 μM; NAC: 10 mM. Calibration bars = 20 μm. (B) Quantification of the effects illustrated in A (Mean ± SEM, N = 3). Statistical significance was determined with one-way ANOVA followed by Tukey multiple comparison test. ***: p <0.001.

Previous studies have established that the RyR1 and RyR2 mammalian isoforms present reactive cysteines that readily undergo redox modifications, such as *S*-glutathionylation, which enhance RyR-mediated CICR [30]. To evaluate if glucose modified RyR2 *S*-glutathionylation levels, we used a novel proximity ligation assay (PLA) that generates a fluorescence signal if the targets lie within an optimal distance of 30–40 nm. In this particular case, the two targets were the RyR2 protein and *S*-glutathionylated protein adducts. Isolated β-cells stimulated for 1 h with 16.7 mM glucose displayed a significant increase in fluorescent dot density (Fig 8A), which increased from a basal value (in arbitrary units) of 37 ± 5 in 2.8 mM glucose, to 129 ± 14 in 16.7 mM glucose (Fig 8B). Incubation of cells with H_2_O_2_ for 1 h induced a similar stimulation of fluorescence intensity (Fig 8A, third row), yielding a fluorescent dot density of 136 ± 15 (Fig 8B). Lastly, β-cells pre-incubated with NAC for 1 h and subsequently stimulated with glucose (16.7 mM) for 1 h displayed a significant reduction of fluorescent dot density (Fig 8A, fourth row), with values of 73 ± 14, dots per cell (Fig 8B). Images of these cells taken at different confocal planes are illustrated in S6 Fig. These results strongly suggest that glucose-induced ROS generation promotes *S*-glutathionylation of RyR2 cysteine residues, which decreases in cells pre-incubated with NAC.

## Discussion

Previous reports have shown that pancreatic islets and β-cell lines express functional RyR channels [9] that give rise to nuclear Ca^2+^ signals [42]. To explore the presence of RyR channels in β-cells, previous studies have employed pharmacological tools [11], endogenous RyR ligands [16, 19, 43], detection of RyR mRNA levels [16, 17, 38] and protein [14, 15, 21, 44], or quantitative determinations of ryanodine binding using fluorescently-labeled ryanodine [38]. Here, we confirmed that β-cells dissociated from pancreatic rat islets and MIN6 pancreatic β-cells express the RyR2 isoform. In contrast to previous studies describing RyR2 localization in insulin secretory vesicles [45] and/or endosomes [21], we found RyR2 co-localized with the ER marker calnexin in both cell types.

### A Role of RyR Channels in GSIS

In spite of the fact that β-cells express functional RyR channels, there is no consensus that RyR-mediated Ca^2+^ release plays a significant role in GSIS [9]. Previous studies have shown that RyR-mediated Ca^2+^ release determines cell viability in pancreatic islets [46] and mediates insulin secretion in INS-1E cells [22]. Additionally, RyR-mediated Ca^2+^ release mediates the activation of TRP-type channels, leading to subsequent depolarization of the plasma membrane [47]; RyR channels also mediate CICR in MIN6 pancreatic β-cells [12] and RyR-mediated Ca^2+^ release contributes to mitochondrial ATP synthesis via GLP-1 [48]. In agreement with previous studies [14, 22], we found that caffeine, which at the low concentrations used in this work acts primarily as a RyR agonist [22], increased GSIS but did not stimulate insulin secretion when added at basal glucose levels. Presumably, activation of RyR-mediated Ca^2+^ release by caffeine does not occur at the resting [Ca^2+^]_i_ and ROS levels present in cells maintained in basal glucose. Furthermore, the antioxidant agent NAC markedly decreased insulin secretion jointly stimulated by glucose and caffeine, suggesting that caffeine requires glucose-induced ROS generation to effectively trigger RyR-mediated CICR and stimulate GSIS.

To examine more directly the role of RyR-mediated Ca^2+^ release on GSIS in pancreatic β-cell islets, we inhibited RyR function with inhibitory concentrations of ryanodine, an agent which to date has no other reported cellular targets. We observed complete GSIS suppression in islets incubated with inhibitory ryanodine for 12 h. This condition did not produce extensive cellular damage, since cholinergic stimulation with CCh of glucose-induced insulin secretion, a process that includes membrane depolarization, InsP_3_ generation, InsP_3_ receptor-mediated Ca^2+^ release and the ensuing fusion of insulin-containing vesicles [39], was not affected. In addition, we show that β-cells retained their ER Ca^2+^ content after prolonged incubation with inhibitory ryanodine, in agreement with a recent report in primary hippocampal neurons [49].

In contrast to the results observed after overnight incubation with ryanodine, we found that exposure of islets for 1 h to inhibitory ryanodine did not affect GSIS. These results are similar to other findings reported in the literature, which provided support for the lack of RyR involvement in GSIS. For example, in isolated human islets, incubation for 1 h with different concentrations of ryanodine (inhibitory and stimulatory) stimulates insulin secretion [21], while 1 h exposure of INS-1 cells to inhibitory ryanodine does not inhibit insulin secretion [22]. Our findings indicate that the exposure time to inhibitory ryanodine is critical to assess the functional roles of RyR in pancreatic islets, and may provide a methodological explanation for the discrepant findings reported in the literature. Based on the slow diffusion of the fluorescent ryanodine analog BODIPY-Ryanodine into the islets, we propose that ryanodine requires a long time to reach inhibitory concentrations in all cells within the islets, which are composed of a highly compact cluster of 1,000–5,000 cells.

### RyR-Mediated GSIS Requires ROS

While ROS are damaging to cells when present in excess, controlled ROS generation plays a central role in cell signaling [50, 51]. Previous reports indicate that β-cells express antioxidant enzymes at relatively low levels [52, 53], a trait which may make β-cells particularly susceptible to oxidative damage. In fact, oxidative stress may be an important factor in the development of β-cell failure during the progression of type-2 diabetes, since excessive ROS production is deleterious for β-cell function [23, 54], and increased ROS production may underlie the cellular damage produced by both lipo- and gluco-toxicicity [23, 55]. Nonetheless, other studies [24, 31] support a role for physiological ROS concentrations as second messengers in insulin secretion. An increase in extracellular glucose concentration enhances ROS generation in pancreatic β-cells [56], as confirmed here, while other studies indicate that GSIS requires mitochondrial ROS production [31]. The low antioxidant enzyme levels of β-cells are likely to make them especially sensitive to ROS-mediated signaling under physiological conditions. Our results, showing that incubation of islets with the antioxidant agent NAC prevented GSIS and markedly decreased insulin secretion jointly stimulated by glucose and caffeine, support and extend these previous findings. NAC has been widely used as an effective antioxidant agent in vivo and in vitro [57]. Results similar to ours have been described in INS-1 cells, where the exogenous application of NAC inhibits insulin secretion stimulated by glucose [24]. We found that NAC did not modify carbachol-stimulated insulin secretion, suggesting that NAC does not prevent alternative cellular mechanisms underlying insulin secretion. Hence, we propose ROS production is a requisite step for GSIS but not for insulin secretion jointly stimulated by glucose and carbachol.

Previous studies in other cell types indicate that RyR channels are highly susceptible to changes in cellular redox state, making RyR a potential cellular redox sensor protein that does not respond to activation by Ca^2+^ when key cysteine residues are in the reduced state [30]. We found that a reduced cellular environment is not conducive to GSIS. Additionally, we observed a direct correlation between GSIS inhibition by NAC and the marked decrease in RyR2 *S*-glutathionylation levels produced by NAC. Consequently, we suggest that GSIS inhibition by NAC is due to reduction of RyR2 cysteine residues, a redox modification that prevents activation of RyR channels by Ca^2+^ in muscle and neurons [55], and that hinders RyR-mediated CICR in other excitable cell types [30]. Supporting our proposal, a recent study in patients with rare RyR2 mutations that produce leaky RyR2 channels, complemented by experiments in islets and β-cells from transgenic mice expressing these defective RyR2 channels (that display intracellular Ca^2+^ leak via oxidized/nitrosylated RyR2 channels), concluded that RyR2 plays a crucial role in the regulation of insulin secretion and glucose homeostasis [58].

### Effects of H_2_O_2_ on Insulin Secretion

Exogenous H_2_O_2_ and diethyl maleate, which increases intracellular H_2_O_2_ levels, stimulate insulin secretion, whereas high concentrations of exogenous antioxidants inhibit GSIS [24]. Our hypothesis predicts that H_2_O_2_-induced insulin secretion at basal glucose concentration involves RyR oxidation, which causes increased RyR-mediated Ca^2+^ release. Our results corroborate this prediction, because both RyR inhibition and NAC prevented insulin secretion induced by H_2_O_2_. Since at basal glucose concentrations H_2_O_2_ enhanced RyR2 *S*-glutathionylation, we propose that this oxidative change contributes to promote RyR-mediated Ca^2+^ release, thereby increasing [Ca^2+^]_i_ to the levels required for insulin secretion. This proposed mechanism is supported by the present results, showing RyR-dependent [Ca^2+^]_i_ increase after addition of H_2_O_2_, as discussed below, and by results obtained in other cell types, where addition of exogenous H_2_O_2_ promotes RyR redox modifications and specifically stimulates RyR-mediated Ca^2+^ release [30, 59].

Additionally, we found that 100 μM H_2_O_2_ disrupted GSIS, confirming previous reports in rat islets [29] and mouse pancreatic β-cells [60]. Chronically high glucose concentrations increase superoxide production and proton leak in mitochondria, reducing ATP levels and causing impaired GSIS in islets from rodents [54]. Hence, we propose that addition of 100 μM H_2_O_2_ in stimulatory glucose produces an abnormal ROS increase and causes oxidative damage, which the weak antioxidant capacity of β-cells presumably fails to neutralize [53], resulting in inhibition of GSIS.

### Effects of H_2_O_2_ on [Ca^2+^]_i_

Thimerosal, an oxidizing agent that effectively enhances the activity of skeletal RyR1 and cardiac RyR2 channels [61], releases Ca^2+^ from InsP_3_-insensitive ER Ca^2+^ pools in RINm5F insulinoma cells and from β-cells isolated from ob/ob mice [62]. Our results show that addition of exogenous H_2_O_2_ to dissociated β-cells maintained in basal glucose increased [Ca^2+^]_i_, which reached values close to 400 nM after H_2_O_2_ addition. These levels are within the range of the [Ca^2+^]_i_ increases elicited by depolarization of human β-cells [63], or elicited by increased glucose levels in cell lines and pancreatic β-cells [9]. This result strengthens our proposal that the increased insulin secretion promoted by H_2_O_2_ at basal glucose concentration is due to an increase in [Ca^2+^]_i_, and extends previous reports showing that H_2_O_2_ increases [Ca^2+^]_i_ to similar levels in islets and β-cell lines through a process that implicates Ca^2+^ release from the ER [29, 64]. A requirement for Ca^2+^ entry has been suggested as well, since removal of extracellular Ca^2+^ suppresses insulin secretion in INS-1 cells in response to H_2_O_2_ [24]. Addition of H_2_O_2_ to rat islets in basal glucose increases [Ca^2+^]_i_ in a dose-dependent manner; this increase is partially sensitive to blockers of L-type channels and is abolished by thapsigargin [65].

In summary, there is consensus that at basal glucose concentration H_2_O_2_ increases [Ca^2+^]_i_ to levels that promote exocytosis of insulin-containing granules, albeit the source of Ca^2+^ remained undefined. Our findings suggest that H_2_O_2_-induced RyR-mediated Ca^2+^ release is a major contributor to the increase in [Ca^2+^]_i_, since H_2_O_2_ did not increase [Ca^2+^]_i_ in cells pre-incubated overnight with inhibitory ryanodine. The present results provide the first evidence that RyR channels are involved in the [Ca^2+^]_i_ increase induced by H_2_O_2_ in β-cells.

## Conclusions

**Fig 9:**
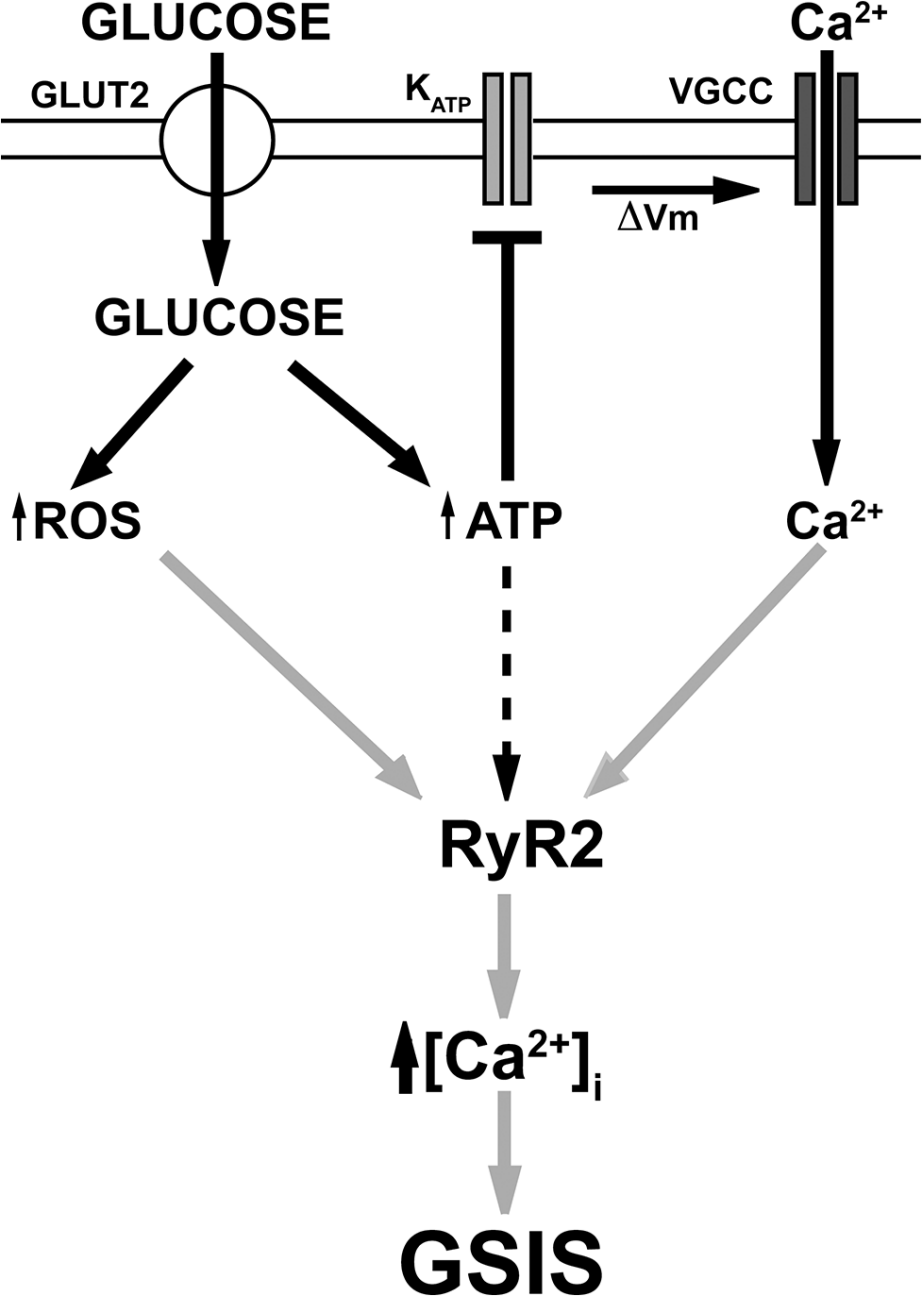
Schematic model of GSIS. Previous studies (thick arrows/lines) have established that an increase in extracellular glucose, the principal physiological insulin secretagogue, stimulates glucose uptake into β-cells via the GLUT-2 transporter. The ensuing accelerated metabolism of intracellular glucose stimulates ROS production and increases the cytoplasmic ATP/ADP ratio. The increase in cytoplasmic ATP promotes closure of ATP-sensitive K^+^ channels (K_ATP_) leading to membrane depolarization and activation of Ca^2+^ influx through voltage-gated Ca^2+^ channels (VGCC). Based on our results, we propose (gray arrows) that the ROS increase induced by glucose promotes RyR2 oxidation (*S*-glutathionylation), which makes possible RyR2-mediated calcium-induced calcium release (CICR) in response to the small and localized [Ca^2+^]_i_ increase produced by Ca^2+^ influx. The glucose-induced ATP increase may also contribute to stimulate CICR mediated by oxidized RyR2 channels (broken arrow). The subsequent [Ca^2+^]_i_ increase promotes insulin exocytosis.

According to the model proposed in this study (Fig 9), the increased ROS generation produced by cellular glucose metabolism makes possible the activation of RyR channels by the local and moderate [Ca^2+^]_i_ increase produced by Ca^2+^ entry from the extracellular medium in response to glucose-induced β-cell depolarization. Although not directly tested here, the glucose-induced increase in ATP concentration may also contribute to enhance RyR channel activation by Ca^2+^, as reported in single RyR channels from neuronal cells [66]. The resulting RyR-mediated CICR would provide the [Ca^2+^]_i_ increase required for insulin secretion. Our hypothesis, presenting GSIS as the combined result of glucose-induced Ca^2+^ entry and glucose-induced ROS generation leading to enhanced RyR-mediated CICR, adds a new concept to the physiology of the pancreatic β-cell. Our results may also explain why prolonged glucose elevations, which promote oxidative stress [67], adversely affect the function of pancreatic β-cells, since excessive activation of RyR-mediated CICR by ROS may promote cellular damage leading to cell death.

## Supporting Information

S1 Fig RyR2 and calnexin immunostaining in MIN6 and pancreatic β-cells. (A) MIN6 cells. Immunostaining directed against RyR2 (green) and the ER marker calnexin (red). The right hand panel illustrates the combined red and green fluorescence plus the blue (Hoechst) nuclear staining. (B) Images were collected from a single pancreatic β-cell. Immunostaining directed against RyR2 (green) and the ER marker calnexin (red). The image at right shows the superposition of green and red fluorescence. Bars indicate 20 μm.
(TIF)

S2 Fig Expression of RyR2 mRNA in rat pancreatic islets and of RyR2 protein in MIN6 cells. (A) RyR2 mRNA was determined by conventional PCR, using the following primer sequences, which are specific for the RyR2 isoform: RyR2sense: 5'-CTACTCAGGATGAGGTCGGA-3'; RyR2antisense: 5'-CTCTCTTCAGATCCAAGCCA-3'. Lane ST: standard; lanes 1, 2, 5 and 6: RNA extracted from rat primary hippocampal neurons. Lanes 3 and 4: RNA extracted from rat pancreatic islets. Lanes 5 and 9: negative controls. The amplified fragment for RyR2 corresponds to 157 bp. (B) RyR2 protein levels in primary hippocampal neurons and MIN6 cells were assayed by Western blot analysis as described in the text.
(TIF)

S3 Fig Distribution of BODIPY-ryanodine. Images were acquired after incubation of pancreatic islets with this probe for 1 h (A) or 12 h (B); both images were obtained by confocal microscopy with identical acquisition parameters, allowing qualitative comparisons. The images at left correspond to fluorescence and at right to transmitted light. Calibration bars: 50 μm.
(TIF)

S4 Fig Ryanodine-treated isolated β-cells displayed similar thapsigargin-elicited Ca^2+^ signals and ROS levels as control cells. (A). Time course of Fluo-4 fluorescence recorded from isolated β-cells before and after addition of thapsigargin to cultures loaded with Fluo-4 AM and transferred to Ca^2+^-free solution just before starting the record. Fluorescence values are expressed as (F/F_0_), where F_0_ represents the basal fluorescence recorded before addition of thapsigargin. Addition of 5 μM thapsigargin (Tg, arrow) elicited similar Ca^2+^ signals in controls (upper panel) as in isolated β-cells pre-incubated with 200 μM ryanodine for 1 h (middle panel) or overnight (bottom panel). (B) Quantification of the areas under the curve. (C) Quantification of maximum fluorescence intensity. In A to C, values represent Mean ± SEM, (N = 3–6 cells from 2 rats). Statistical significance was determined with one-way ANOVA followed by Tukey's multiple comparison test. ns: no significant differences. (D). Representative fluorescence images (upper) of islets loaded with 10 μM CM-H_2_DCFDA, collected by confocal microscopy; at bottom, light-contrast images. (E) Quantification of H_2_DCFDA fluorescence intensity determined in control islets, in islets pre-incubated with 200 μM ryanodine for 1 h or overnight, or treated with 0.5 mM H_2_O_2_ for 1 h. N = 4–10 islets. ***: p < 0.001, determined by statistical analysis with One-way ANOVA, followed by Tukey’s post-hoc test.
(TIF)

S5 Fig N-acetyl cysteine (NAC) does not prevent insulin secretion induced by carbachol. The effects of NAC were tested in either basal (2.8 mM) or stimulatory (27.7 mM) glucose (G) concentrations. Values represent Mean ± SEM, N = 3. Statistical significance was determined with one-way ANOVA followed by Tukey's Multiple Comparison Test. *: p <0.05; ***: p <0.001; ns: no significant differences.
(TIF)

S6 Fig Determination of RyR2 *S*-glutathionylation with the PLA assay. The figure displays representative confocal images acquired in disaggregated β-cells from islets, showing PLA labeling (red), insulin immunostaining (green) and the merged images. From left to right, images were taken at different depths, from the bottom to the top of cells incubated in basal glucose (2.8 mM), stimulatory glucose (16.7 mM), basal glucose (2.8 mM) plus H_2_O_2_ (100 μM) or stimulatory glucose (16.7 mM) plus NAC (10 mM).
(JPG)
