# Correction: Glucose-Dependent Insulin Secretion in Pancreatic β-Cell Islets from Male Rats Requires Ca^2+^ Release via ROS-Stimulated Ryanodine Receptorsmographic and Clinico-Epidemiological Features of Dengue Fever in Faisalabad, Pakistan

**DOI:** 10.1371/journal.pone.0140198

**Published:** 2015-10-02

**Authors:** Paola Llanos, Ariel Contreras-Ferrat, Genaro Barrientos, Marco Valencia, David Mears, Cecilia Hidalgo

The following information is missing from the Funding section: This study was supported by FONDECYT 1140545, CONICYT-Chile (http://spl.conicyt.cl/auth/) to CH.
