# Correction: Correction: Glucose-Dependent Insulin Secretion in Pancreatic β-Cell Islets from Male Rats Requires Ca^2+^ Release via ROS-Stimulated Ryanodine Receptorsmographic and Clinico-Epidemiological Features of Dengue Fever in Faisalabad, Pakistan

**DOI:** 10.1371/journal.pone.0143039

**Published:** 2015-11-10

**Authors:** The PLOS ONE Staff

There is an error in the title of the correction published October 2, 2015. The correct title is: Correction: Glucose-Dependent Insulin Secretion in Pancreatic β-Cell Islets from Male Rats Requires Ca^2+^ Release via ROS-Stimulated Ryanodine Receptors. The publisher apologizes for the error.
