# Supplementary figures and images for: Glucose-Dependent Insulin Secretion in Pancreatic β-Cell Islets from Male Rats Requires Ca2+ Release via ROS-Stimulated Ryanodine Receptors
Source: PLoS One. 2015 Jun 5;10(6):e0129238. doi: 10.1371/journal.pone.0129238 (PMC4457734; doi:10.1371/journal.pone.0129238)

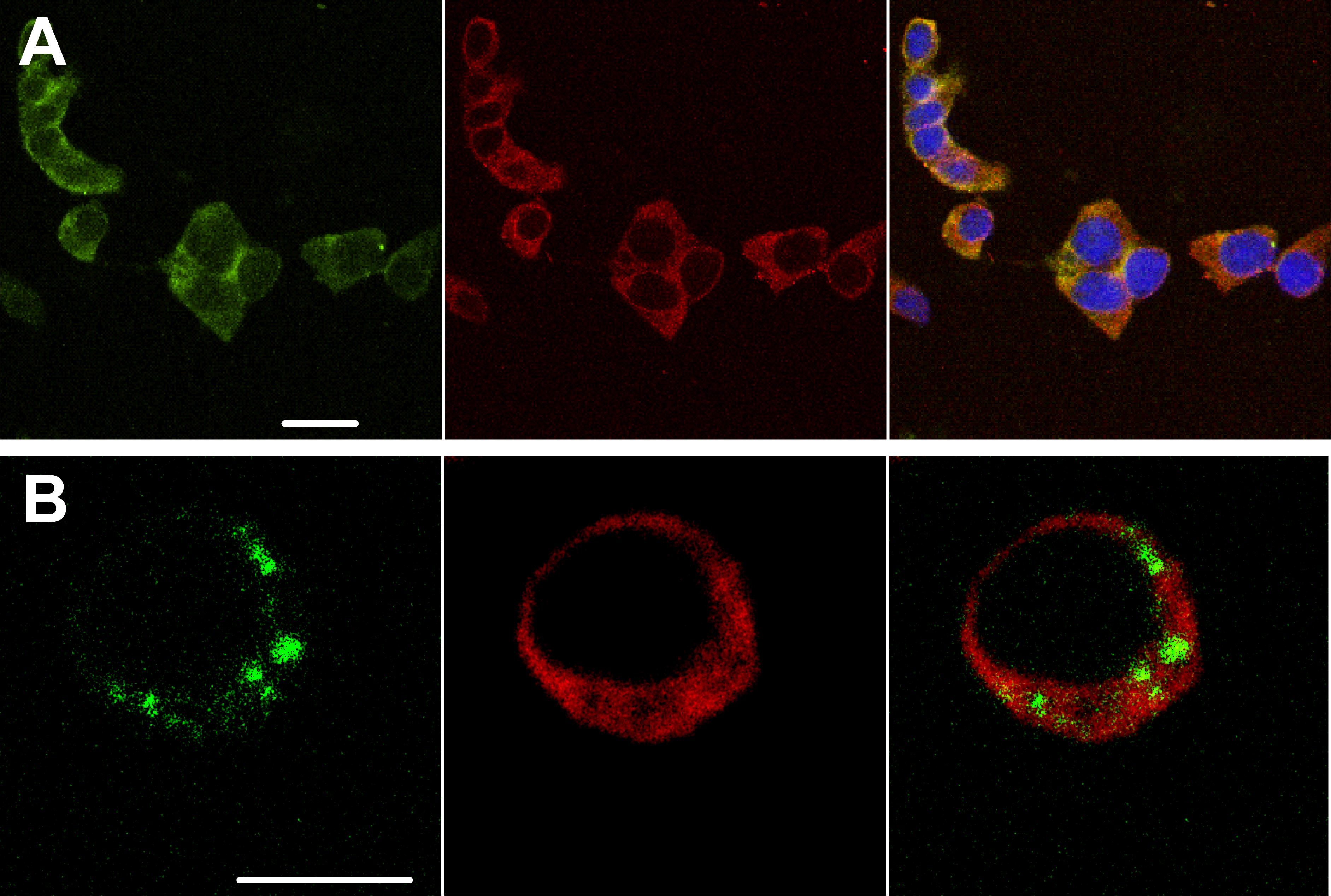

Supplement: S1 Fig — (A) MIN6 cells. Immunostaining directed against RyR2 (green) and the ER marker calnexin (red). The right hand panel illustrates the combined red and green fluorescence plus the blue (Hoechst) nuclear staining. (B) Images were collected from a single pancreatic β-cell. Immunostaining directed against RyR2 (green) and the ER marker calnexin (red). The image at right shows the superposition of green and red fluorescence. Bars indicate 20 μm. (TIF) [file pone.0129238.s001.tif]

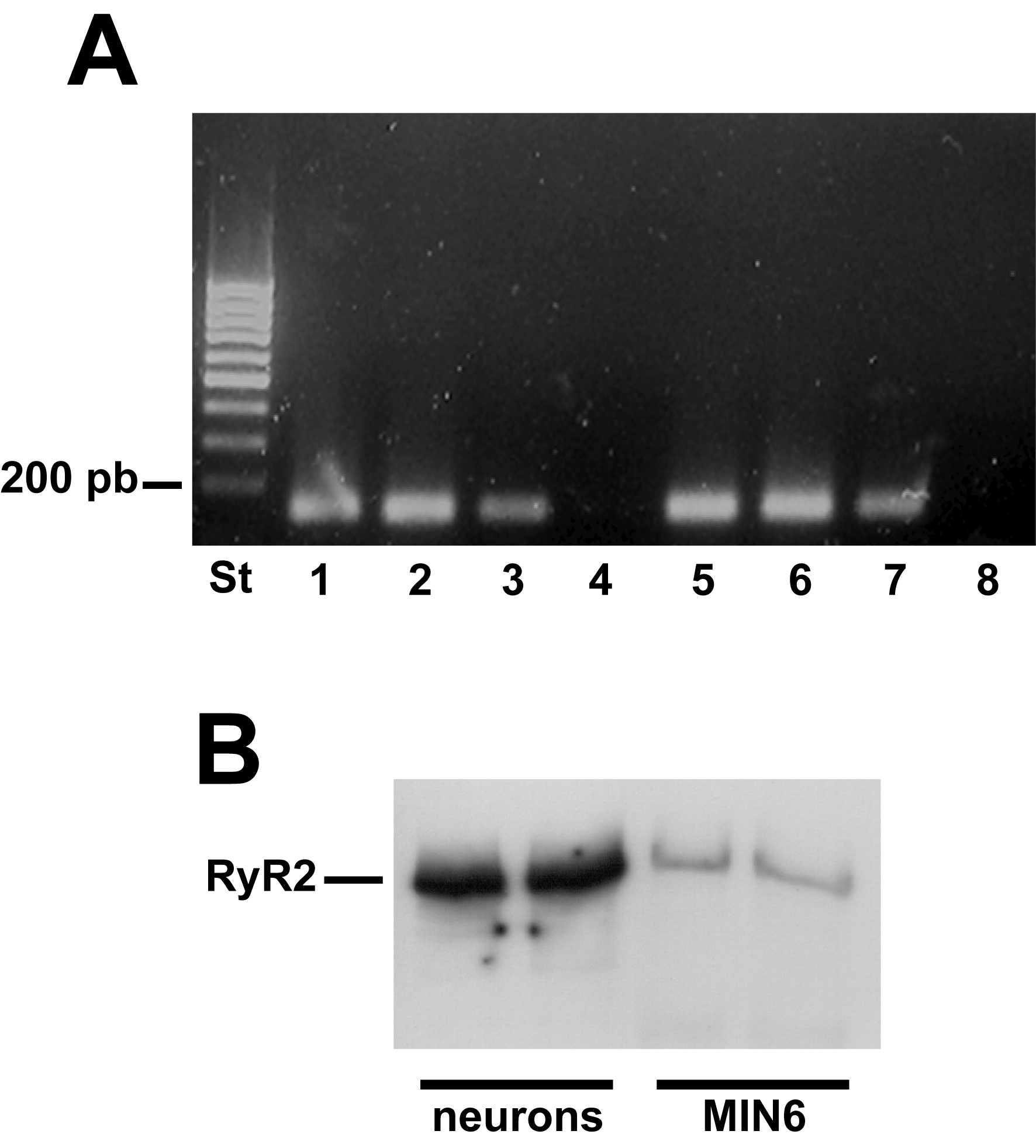

Supplement: S2 Fig — (A) RyR2 mRNA was determined by conventional PCR, using the following primer sequences, which are specific for the RyR2 isoform: RyR2sense: 5'-CTACTCAGGATGAGGTCGGA-3'; RyR2antisense: 5'-CTCTCTTCAGATCCAAGCCA-3'. Lane ST: standard; lanes 1, 2, 5 and 6: RNA extracted from rat primary hippocampal neurons. Lanes 3 and 4: RNA extracted from rat pancreatic islets. Lanes 5 and 9: negative controls. The amplified fragment for RyR2 corresponds to 157 bp. (B) RyR2 protein levels in primary hippocampal neurons and MIN6 cells were assayed by Western blot analysis as described in the text. (TIF) [file pone.0129238.s002.tif]

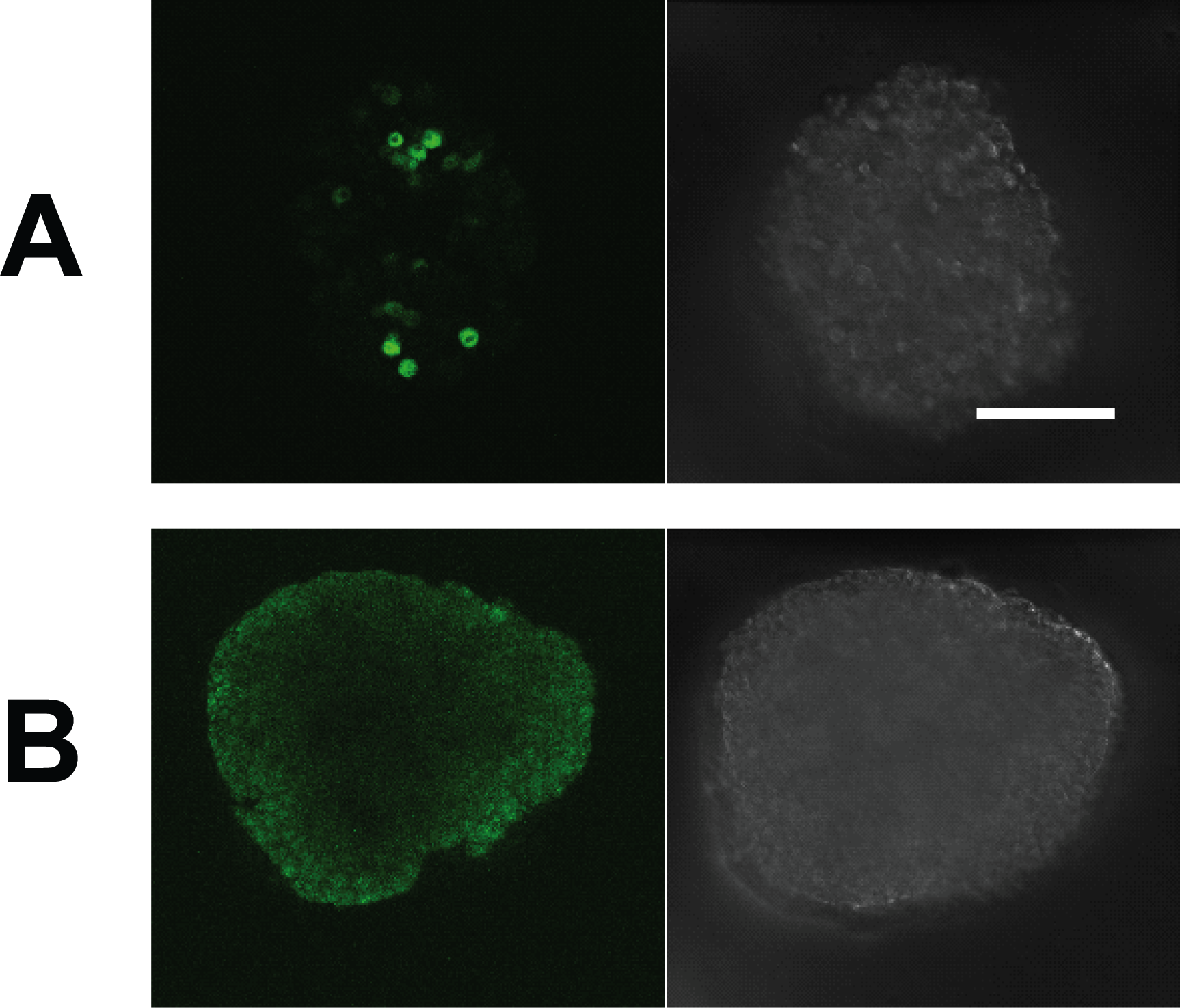

Supplement: S3 Fig — Images were acquired after incubation of pancreatic islets with this probe for 1 h (A) or 12 h (B); both images were obtained by confocal microscopy with identical acquisition parameters, allowing qualitative comparisons. The images at left correspond to fluorescence and at right to transmitted light. Calibration bars: 50 μm. (TIF) [file pone.0129238.s003.tif]

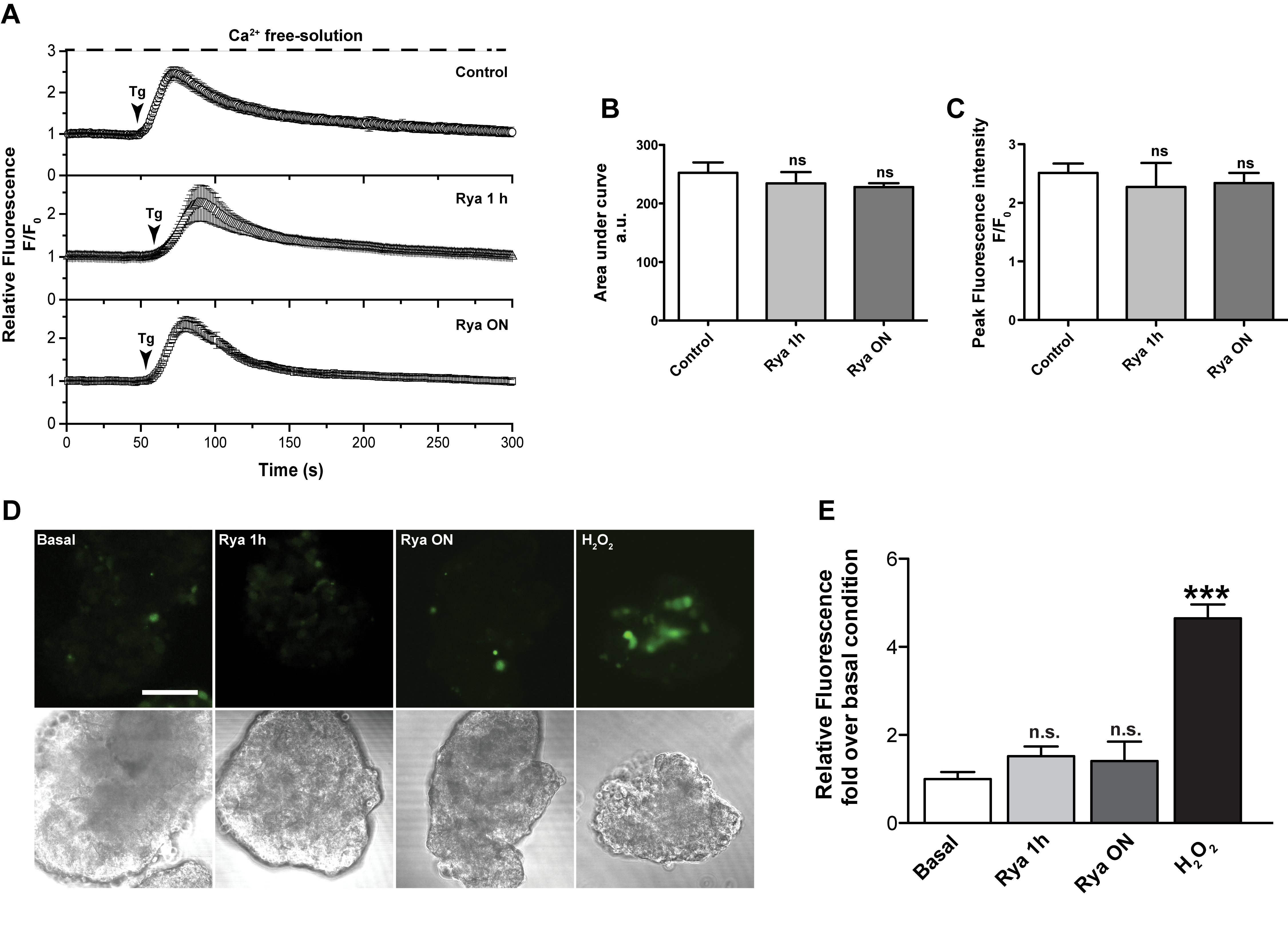

Supplement: S4 Fig — (A). Time course of Fluo-4 fluorescence recorded from isolated β-cells before and after addition of thapsigargin to cultures loaded with Fluo-4 AM and transferred to Ca2+-free solution just before starting the record. Fluorescence values are expressed as (F/F0), where F0 represents the basal fluorescence recorded before addition of thapsigargin. Addition of 5 μM thapsigargin (Tg, arrow) elicited similar Ca2+ signals in controls (upper panel) as in isolated β-cells pre-incubated with 200 μM ryanodine for 1 h (middle panel) or overnight (bottom panel). (B) Quantification of the areas under the curve. (C) Quantification of maximum fluorescence intensity. In A to C, values represent Mean ± SEM, (N = 3–6 cells from 2 rats). Statistical significance was determined with one-way ANOVA followed by Tukey's multiple comparison test. ns: no significant differences. (D). Representative fluorescence images (upper) of islets loaded with 10 μM CM-H2DCFDA, collected by confocal microscopy; at bottom, light-contrast images. (E) Quantification of H2DCFDA fluorescence intensity determined in control islets, in islets pre-incubated with 200 μM ryanodine for 1 h or overnight, or treated with 0.5 mM H2O2 for 1 h. N = 4–10 islets. ***: p < 0.001, determined by statistical analysis with One-way ANOVA, followed by Tukey’s post-hoc test. (TIF) [file pone.0129238.s004.tif]

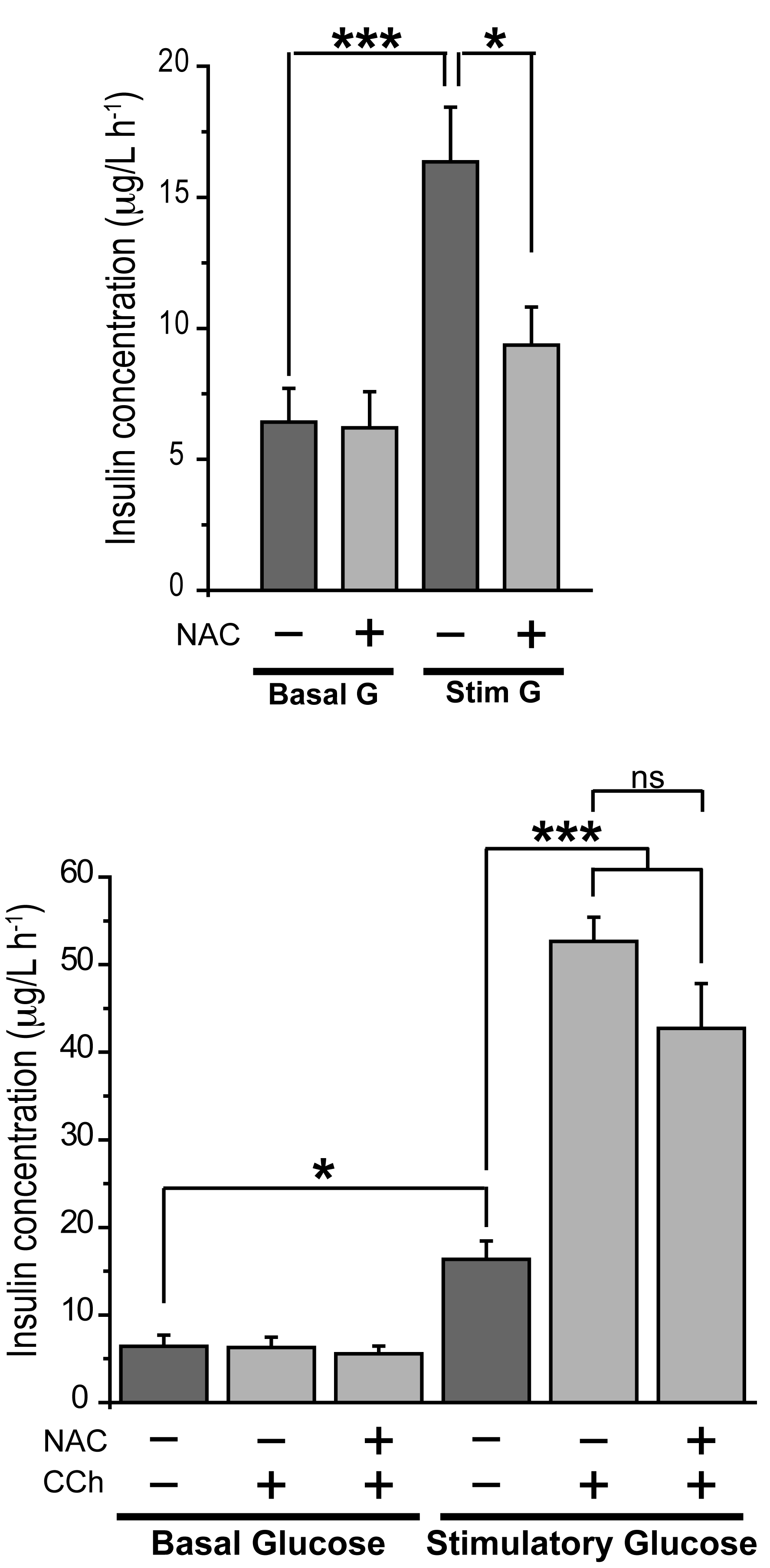

Supplement: S5 Fig — The effects of NAC were tested in either basal (2.8 mM) or stimulatory (27.7 mM) glucose (G) concentrations. Values represent Mean ± SEM, N = 3. Statistical significance was determined with one-way ANOVA followed by Tukey's Multiple Comparison Test. *: p <0.05; ***: p <0.001; ns: no significant differences. (TIF) [file pone.0129238.s005.tif]

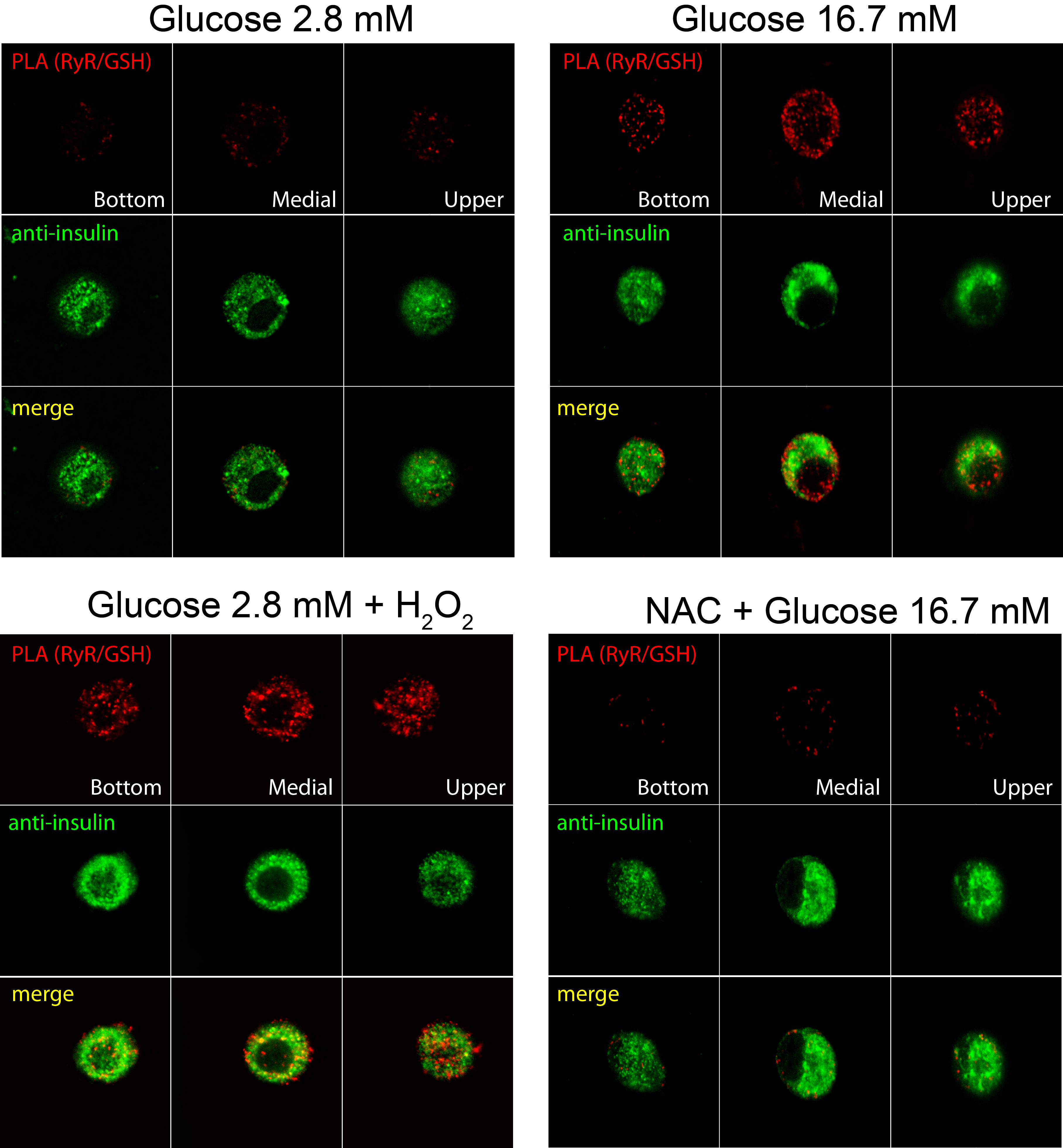

Supplement: S6 Fig — The figure displays representative confocal images acquired in disaggregated β-cells from islets, showing PLA labeling (red), insulin immunostaining (green) and the merged images. From left to right, images were taken at different depths, from the bottom to the top of cells incubated in basal glucose (2.8 mM), stimulatory glucose (16.7 mM), basal glucose (2.8 mM) plus H2O2 (100 μM) or stimulatory glucose (16.7 mM) plus NAC (10 mM). (JPG) [file pone.0129238.s006.jpg]
